# Identification of key biomarkers and immune infiltration in the co-occurrence of oral lichen planus and Hashimoto’s thyroiditis by integrated bioinformatics analysis

**DOI:** 10.1097/MD.0000000000046324

**Published:** 2026-05-12

**Authors:** Hui Feng, Zhen Chen, Li Xiao

**Affiliations:** a Beijing Anzhen Nanchong Hospital, Capital Medical University & Nanchong Central Hospital, Nanchong, China.

**Keywords:** bioinformatics analysis, Hashimoto thyroiditis, immune infiltration, oral lichen planus

## Abstract

Increasing evidence have suggested the co-occurrence of oral lichen planus (OLP) and Hashimoto thyroiditis (HT), the underlying mechanism remains unclear. The aim of our study was to investigate co-expressed differentially expressed genes (co-DEGs) of OLP and HT, elucidate the mechanisms responsible for co-occurrence of OLP and HT. The expression profiles of GSE52130 and GSE29315 were downloaded from the Gene Expression Omnibus database to obtain DEGs between disease group and healthy control group by GEO2R. Enrichment analysis of co-DEGs were performed by Metascape platform. The protein–protein interaction (PPI) network of co-DEGs were developed utilizing the STRING database. Subsequently, CIBERSORT was utilized to evaluate the infiltration of immune cells in OLP and HT. Finally, enzyme-linked immunosorbent assay and quantitative real-time PCR were conducted to verify the key genes in clinic samples. A total of 76 co-DEGs were recognized. Enrichment analysis demonstrated that these co-DEGs focused primarily on cytokine signaling in immune system. The 10 most firmly related genes among co-DEGs were identified from the PPI network. We found that immune cell infiltration associated with VCAM1 expression was correlated with OLP and HT. The top 3 co-DEGs (CXCL12, CXCL10, and VCAM1) in PPI network were verified in OLP and HT samples. VCAM1 may be closely related pathogenesis of co-occurrence of OLP and HT, and represents a new candidate molecular marker of the occurrence and progression of OLP and HT. Moreover, immune cell infiltration associated with VCAM1 expression plays an important role in the coexistence of OLP and HT.

## 1. Introduction

Oral lichen planus (OLP) is a persistent inflammatory condition predominantly mediated by immune responses, and it has been observed to occur more frequently in women than in men. Recent epidemiological studies estimate its prevalence in the general population to range between 1.27% and 2.0%.^[1]^ OLP has been classified by the World Health Organization as a potentially malignant condition affecting the oral mucosa, with studies reporting a transformation rate to malignancy ranging between 0.4% and 12.5%.^[2,3]^ The precise cause of OLP remains unclear. However, it is widely regarded as a multifactorial condition influenced by a range of contributing factors, including mechanical irritation, electrochemical stimuli, trauma, psychological stress, infections, nutritional deficiencies, allergic reactions, endocrine imbalances, genetic predisposition, and immune-related disorders.^[4]^ Although the underlying cause of OLP has yet to be fully elucidated, immune system dysfunction is believed to be a key contributor to its onset and progression. Local immune responses, particularly those mediated by CD4 + and CD8 + T lymphocytes, are thought to play a significant role in the pathogenesis of OLP.^[5,6]^

Hashimoto thyroiditis (HT), is considered to be the most common autoimmune disease, which is characterized by thyroid-specific autoantibodies.^[7]^ The pathology is diagnosed 5 to 10 times more often in women than men and its incidence increase with the age.^[8]^ HT is pathologically characterized by lymphocytic infiltration, especially of T cells, and follicular destruction are the histological hallmark of HT, that lead to progressive atrophy and fibrosis.^[9]^ Clinically, the mainly feature of HT is systemic manifestations due to the damage of the thyroid gland, developing a primary hypothyroidism.^[10]^ Current studies indicates that HT coexists with or is associated with some other systemic diseases, especially some autoimmune diseases, such as systemic lupus erythematosus,^[11]^ autoimmune encephalitis,^[12]^ Sjögren’s syndrome,^[13]^ and OLP.^[14]^

Kurgansky et al^[15]^ reported the first case of coexisting lichen planus and hypothyroidism in 1994. Since then, the possible relationship between thyroid disease and OLP have attracted more and more attention. Wu et al^[16]^ found that there is a common or overlapping pathogenesis in terms of immune, heredity, environmental, and hormonal factors between OLP and HT, which might cause co-occurrence. While an increasing number of studies have investigated the possible relationship between HT and OLP,^[17–19]^ the underlying mechanism remains unclear. In our study, we identified co-expressed differentially expressed genes (co-DEGs) of OLP and HT by bioinformatic analysis and elucidated molecular mechanisms and pathology of OLP-related DEGs (OLP-DEGs) and HT-related DEGs (HT-DEGs). Finally, we provide some preliminary insights into the possible association between OLP and HT pathogenesis.

## 2. Materials and methods

### 2.1. Tissue samples and bioinformatics analysis

RNA expression data for tissue samples were obtained from the Gene Expression Omnibus (GEO) database (http://www.ncbi.nlm.nih.gov/geo/). The dataset GSE52130 contained 7 specimens from OLP patients and 7 normal controls, with expression profiling arrays generated using the GPL10558 platform (Illumina HumanHT-12 V4.0 expression beadchip). Another dataset, GSE29315, comprised 6 specimens from HT patients and 8 controls, with expression profiling arrays generated on the GPL8300 platform (Affymetrix Human Genome U95 Version 2 Array).

### 2.2. Identification of differentially expressed genes (DEGs)

We utilized GEO2R (http://www.ncbi.nlm.nih.gov/geo/geo2r), an online tool, to identify DEGs between OLP/HT samples and normal controls. The criteria for DEG identification were |log2FoldChange (FC)| > 1.0 and an adjusted *P*-value < .05. The results were visualized using the “ggplot2” package in R software (R Foundation for Statistical Computing, Vienna, Austria) to generate heatmaps and volcano plots related to OLP- and HT-DEGs. Venn diagrams were created using the Venn online tool (http://bioinformatics.psb.ugent.be/webtools/Venn/), and the co-expression DEGs from both OLP and HT were selected for further analysis.

### 2.3. Enrichment analysis of co-expression DEGs

We performed enrichment analysis on the co-expression DEGs using the Metascape platform (https://metascape.org/gp/index.html), which provides comprehensive gene annotation. The biological processes and pathways of co-expressed DEGs were analyzed and visualized using bubble diagrams created with the “ggplot2” package in R software.

### 2.4. Protein–protein interaction (PPI) network and module analysis

PPI data were retrieved from the STRING database (www.string-db.org), a widely used resource for exploring protein interactions. We constructed the PPI network and visualized it using Cytoscape software (version 3.7.1; Cytoscape Consortium, San Diego). To identify highly interconnected regions within the network, we applied the molecular complex detection (MCODE) plugin, using the following parameters: MCODE score > 3, degree threshold ≥ 2, node score cutoff ≥ 0.2, and a maximum depth of 100. The top 10 nodes based on their centrality values were considered as key hub genes.

### 2.5. Evaluation of immune cell infiltration

Immune cell infiltration in OLP and HT samples was assessed using the CIBERSORT algorithm (https://cibersort.stanford.edu/), which calculates the proportions of 22 types of leukocytes. A significance threshold of *P* < .05 was applied. The immune cell composition in each sample was visualized using bar plots and heatmaps. Correlations between immune cell infiltration and vascular cell adhesion molecule 1 (VCAM1) expression were also analyzed and visualized with the “ggplot2” package in R software.

### 2.6. Sample collection

Blood and tissue samples were collected from patients and healthy controls at Nan Chong Central Hospital (Nanchong, China), with written informed consent obtained from all participants. The research protocol was approved by the Ethics Committee of North Sichuan Medical College (approval number:2020‐2020‐25‐2).

### 2.7. Enzyme-linked immunosorbent assay (ELISA)

Serum levels of CXCL12, CXCL10, and VCAM1 were measured using ELISA kits (Abcam, Cambridge, UK) in accordance with the manufacturer’s instructions. Blood samples were obtained between 2021 and 2022 and stored at −80°C prior to analysis. Absorbance at 450 nm was measured using a microplate reader, and the concentration of each sample was determined using a standard curve generated from serial dilutions of standards.

### 2.8. Quantitative real-time PCR (qPCR)

Total RNA was extracted from tissue samples obtained between 2021 and 2022, stored at −80°C, and reverse transcribed to cDNA using the PrimeScript RT Reagent Kit (TaKaRa, Japan). qPCR was performed using SYBR Green (Roche). Primer sequences for CXCL12, CXCL10, and VCAM1, along with GAPDH as the internal control, were as follows:

CXCL12: Forward: 5′-ATTCTCAACACTCCAAACTGTGC-3′, Reverse: 5′-ACTTTAGCTTCGGGTCAATGC-3′.

CXCL10: Forward: 5′-GTGGCATTCAAGGAGTACCTC-3′, Reverse: 5′-GTGGCATTCAAGGAGTACCTC-3′.

VCAM1: Forward: 5′-GGGAAGATGGTCGTGATCCTT-3′, Reverse: 5′-TCTGGGGTGGTCTCGATTTTA-3′.

GAPDH: Forward: 5′-GACTCATGACCACAGTCCATGC-3′, Reverse: 5′-AGAGGCAGGGATGATGTTCTG-3′.

Relative expression levels of CXCL12, CXCL10, and VCAM1 were normalized to GAPDH and calculated using the 2^−△△CT^ method.

### 2.9. Statistical analysis

Statistical analyses were performed using R software (version 4.0.0) and SPSS software (version 25.0; IBM Corp., Armonk). For comparisons between 2 groups, the Student *t* test was employed for normally distributed data, and the Mann–Whitney *U* test was used for non-normally distributed data. The significance threshold was set at *P* < .05. DEGs were identified using GEO2R with a cutoff of |log2FC| > 1.0 and an adjusted *P*-value < .05. The correlation between immune cell infiltration and VCAM1 expression was evaluated using Pearson correlation analysis. Enrichment analyses were performed using the Metascape platform, and the PPI network was visualized using Cytoscape (version 3.7.1). Venn diagrams and heatmaps were generated using the “Venn” online tool and the “ggplot2” package in R software.

## 3. Results

### 3.1. Identification of DEGs

**Figure 1.**
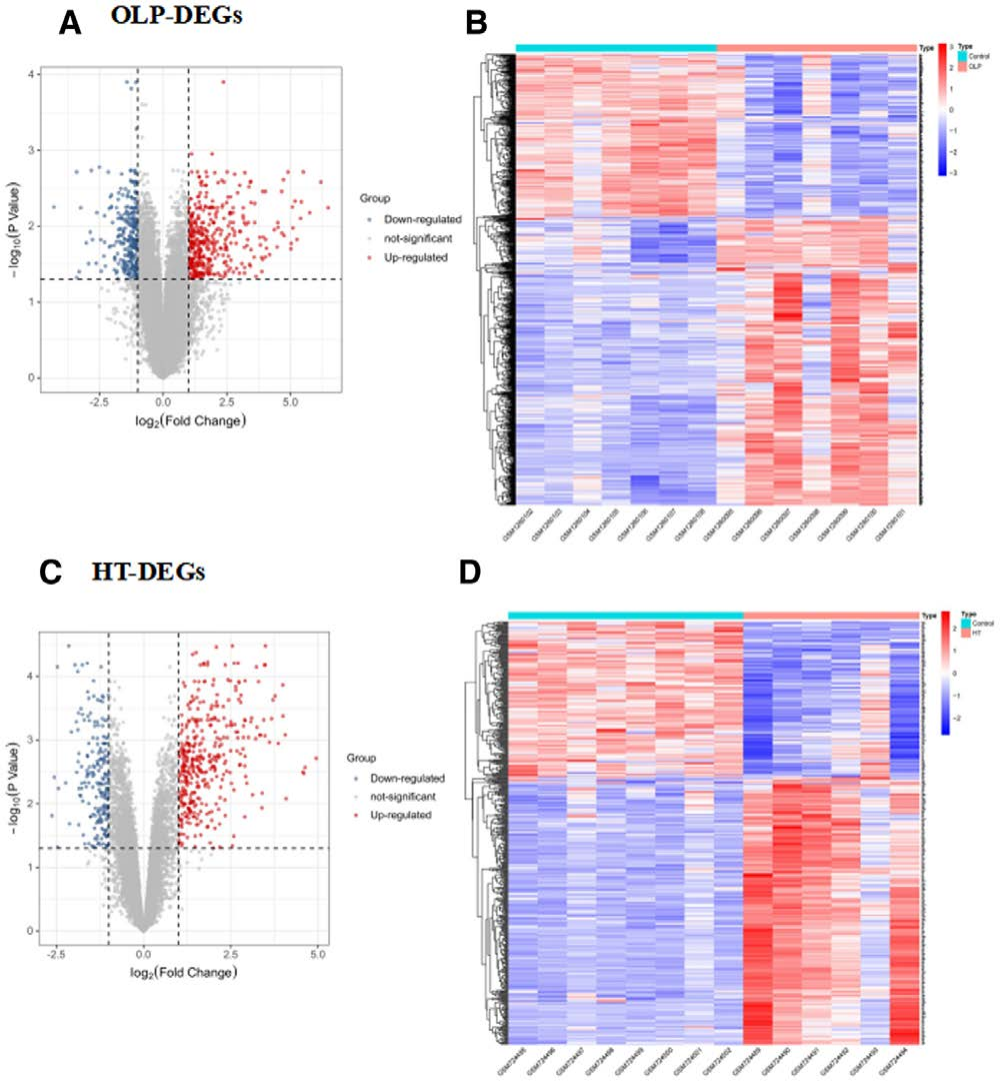
Identification of DEGs. DEGs were identified from GSE52130 (OLP), hierarchical clustering (A) and (B) volcano plot. DEGs were identified from GSE29315 (HT), hierarchical clustering (C) and (D) volcano plot. The black dots in the figure represent genes that were not differentially expressed, the red dots represent upregulated DEGs, and the green points represent downregulated DEGs. DEGs, differentially expressed genes. Significant gene sets were |fold change|≥ 1 and adjusted *P* value < .05. DEGs = differentially expressed genes, HT = Hashimoto thyroiditis, OLP = oral lichen planus.

In GSE52130, a differential gene expression analysis was performed using the dataset that included 7 OLP samples and 7 control oral epithelium samples. We identified 814 DEGs based on thresholds of adjusted *P*-value < .05 and |log2FC| >1 as the standard in OLP patients compared with control species, including 294 down-regulated genes and 520 up-regulated genes, as is shown in the volcano map and heat map (Fig. 1A–B). However, in GSE29315, total of 529 DEGs were screened in 6 HT patients compared with 8 control species, including 194 down-regulated genes and 335 up-regulated genes, as is shown in the volcano map and heat map (Fig. 1C–D).

### 3.2. Functional enrichment analysis of co-expression DEGs

**Figure 2.**
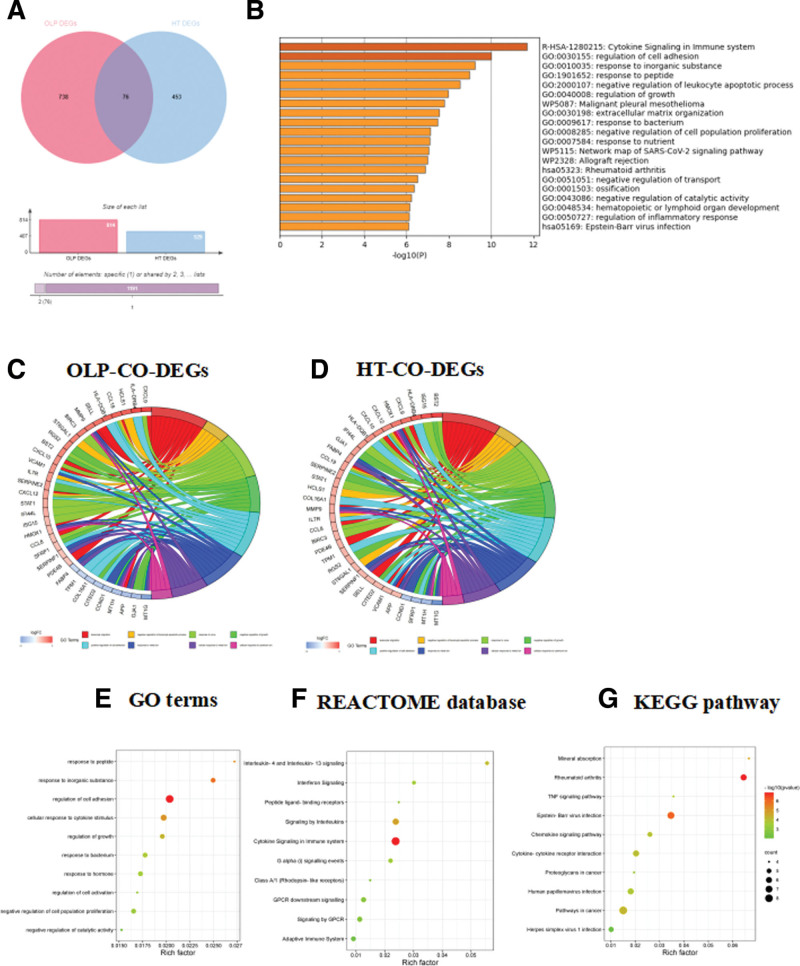
Functional enrichment analysis of co-expression DEGs. (A) Venn diagram was conducted to obtain the intersection of the DEGs screened from GSE52130 (OLP) and GSE29315 (HT). (B) Enrichment analysis of co-DEGs in Metascape, co-DEGs, co-expression DEGs. Enriched terms are colored by *P*-value, where terms containing more genes tend to have more significant *P*-value. Gene ontology (GO) enrichment analysis of co-DEGs in GSE52130 (OLP) (C) and GSE29315 (HT) (D). Co-DEGs were enriched in GO enrichment analysis (E), REACTOME database (F), and KEGG pathway (G), and the top 10 most significant pathway were shown. Dot sizes represent counts of enriched DEGs, and dot colors represent negative Log10-*P* values. DEGs = differentially expressed genes, HT = Hashimoto thyroiditis, KEGG = Kyoto Encyclopedia of Genes and Genomes, OLP = oral lichen planus.

The intersection of 814 OLP-DEGs and 529 HT-DEGS was taken, which found 76 co-DEGs (Fig. 2A). All 76 co-DEGs were classified using Metascape database. As shown in Table S1, Supplemental Digital Content, https://links.lww.com/MD/Q833 and Figure 2B, co-DEGs were predominantly enriched in the process of cytokine signaling in immune system (22.37%) and regulation of cell adhesion (21.05%), which showed that the co-DEGs were mainly involved in immune pathways. Gene ontology enrichment analysis revealed that the co-DEGs were significantly enriched in leukocyte migration, negative regulation of leukocyte apoptotic process and response to virus in OLP and HT samples (Fig. 2C–D). The data showed that co-DEGs were mainly enriched in regulation of cell adhesion, regulation of cell adhesion and response to inorganic substance in gene ontology biological processes (Fig. 2E). As for the Reactome gene sets, co-DEGs were largely involved in cytokine signaling in immune system, signaling by Interleukins, and GPCR downstream signaling (Fig. 2F). In the Kyoto Encyclopedia of Genes and Genomes database, the co-DEGs were predominantly enriched in the pathways in cancer, Epstein-Barr virus infection and cytokine–cytokine receptor interaction (Fig. 2G).

### 3.3. PPI network and module analysis

**Figure 3.**
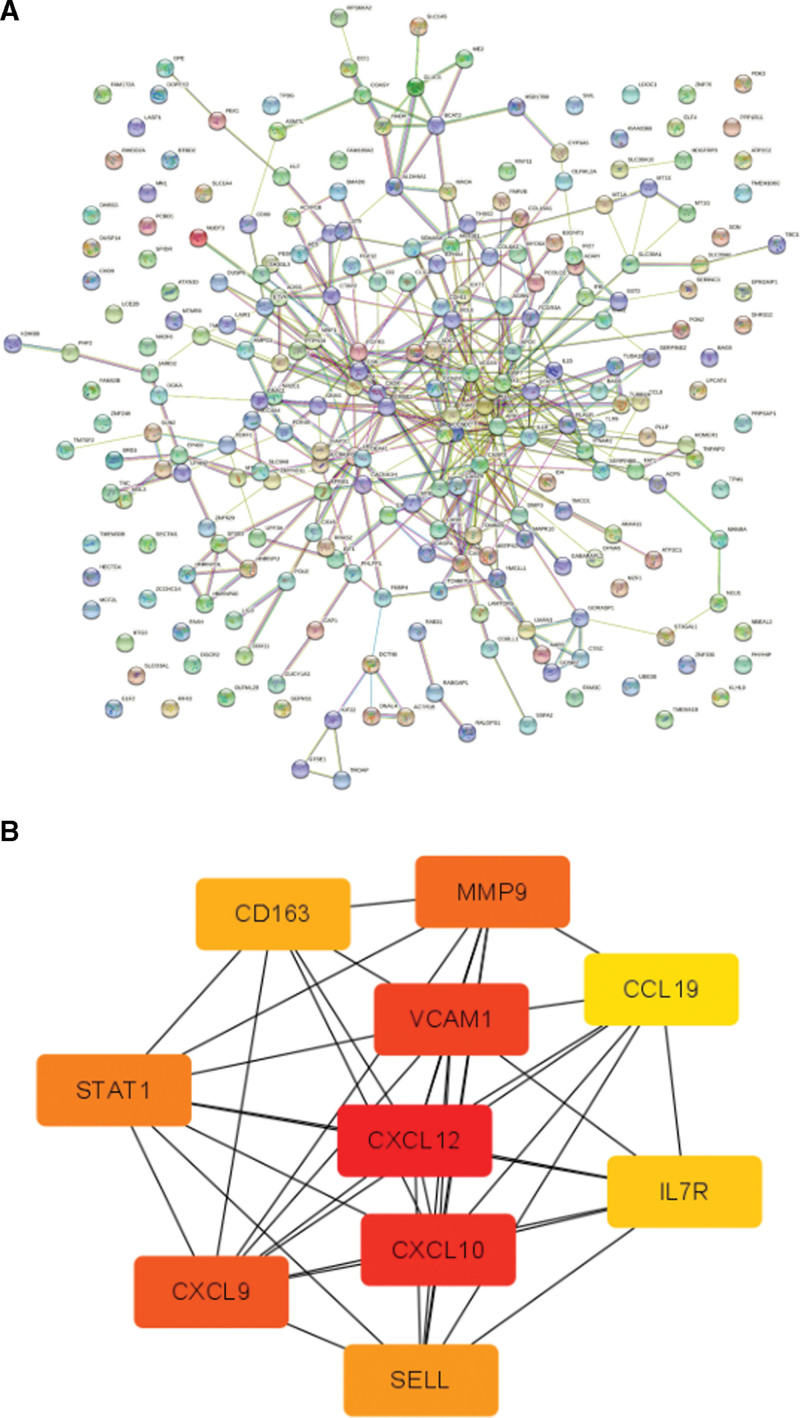
PPI network (A) and module analysis (B). The PPI network of DEGs was constructed using Cytoscape. Identification of a subnetwork using MCODE in Cytoscape software. The top 10 key genes were screened through the PPI network map. The different colors in the image merely represent different genes and have no other substantial meaning. DEGs = differentially expressed genes, PPI = protein–protein interaction.

To further screen out hub genes, the PPI network of the co-DEGs was constructed based on the information obtained from the STRING database. When 76 co-DEGs were submitted to the STRING database, there were 1 unidentified gene IDs. Subsequently, 75 genes were mapped into the PPI network (Fig. 3A). A centrality analysis of the nodes in the PPI network revealed that C-X-C motif chemokine ligand 12 (CXCL12), C-X-C motif chemokine ligand 10 (CXCL10), vascular cell adhesion molecule 1 (VCAM1), C-X-C motif chemokine ligand 9 (CXCL9), matrix metallopeptidase 9 (MMP9), signal transducer and activator of transcription 1 (STAT1), selectin L (SELL), CD163, interleukin 7 receptor (IL7R), and C-C motif chemokine ligand 19 (CCL19) were crucial genes by cytoscape and cytoHubba analysis (Fig. 3B). Interestingly, these 10 genes were up-regulated in both OLP and HT. Using the Reactome database, the top 10 genes were primarily associated with inflammatory response, immune response, regulation of NF-kappa-B signaling, MAPK6/MAPK4 signaling, and modulates innate and adaptive immune responses.

### 3.4. Correlation of VCAM1 with immune infiltration in OLP and HT

In the enrichment analysis, our results indicated that 22 co-DEGs were enriched in immune pathway, which was the pathway with the most genes among all the enriched pathways. Therefore, based on the results of previous studies and our bioinformatic analysis, we speculate that the pathogenesis of both diseases is most related to immunity.

**Figure 4.**
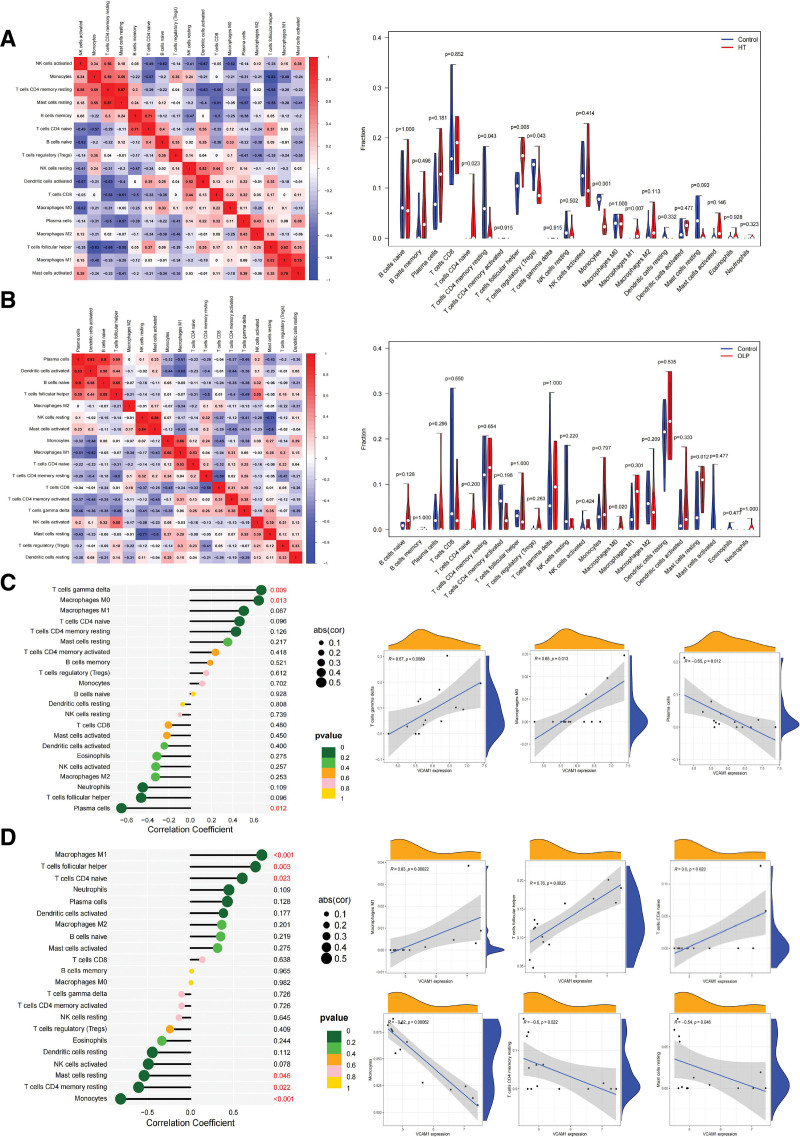
Correlation of VCAM1 with immune infiltration in OLP and HT. Heatmap showed the correlation between 22 kinds of immune cells in HT (A) and OLP (B). The size of the colored squares represents the strength of the correlation. Red represents a positive correlation, blue represents a negative correlation. The darker the color, the stronger the correlation. Violin diagram of the proportion of 22 types of immune cells. A GSE29315 (HT), B GSE51130 (OLP), showed the difference in infiltration between the 2 groups. Correlation of immune infiltration with VCAM1 expression in OLP (C) and HT (D). Scatter plot showed the correlation of immune cells proportion with the VCAM1 expression (*P* < .05). The blue line in each plot was fitted linear model indicating the proportion tropism of the immune cell along with VCAM1 expression. HT = Hashimoto thyroiditis, OLP = oral lichen planus.

Previous studies indicated that in the top 3 genes, VCAM1 was involved in both lymphocyte activation in OLP and autoimmune response in HT.^[20,21]^ To further confirm the interactions between VCAM1 expression and immune infiltration, the CIBERSORT algorithm was used to analyze the proportion of 22 immune cell types in OLP and HT. As shown in Figure 4A, the HT samples tended to have a lower proportion of regulatory T cell, T cell CD4 naïve and monocytes, and a higher proportion of T cells follicular helper, T cells CD4 memory resting and macrophages M1. In Figure 4B, the OLP samples tended to have a higher proportion of macrophages M0 and mast cells resting. Meanwhile, the difference and correlation analysis of OLP and HT samples also revealed that the infiltrating immune cells were significantly associated with VCAM1 expression levels. In OLP samples, T cells gamma delta and macrophages M0 were positively correlated with VCAM1 expression, whereas Plasma cells were negatively associated with VCAM1 expression (Fig. 4C). In HT samples, macrophages M1, T cells follicular helper, and T cells CD4 naive were positively correlated with VCAM1 expression, whereas monocytes, T cells CD4 memory resting and mast cells resting were negatively associated with VCAM1 expression (Fig. 4D). These results indicated that VCAM1 might participate in the co-occurrence of OLP and HT through modulation of immune cell infiltration levels.

### 3.5. Verification of potential experimental target expression

**Figure 5.**
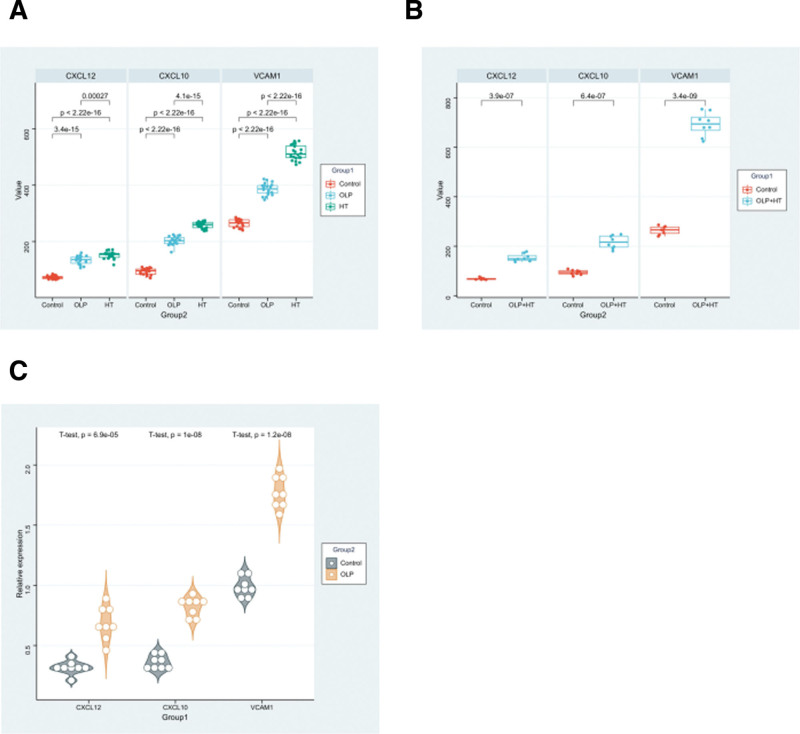
Verification of potential experimental target expression. (A) Serum levels of CXCL12, CXCL10, and VCAM1 in patients with OLP and HT in comparison with healthy controls by ELISA. (B) Serum levels of CXCL12, CXCL10, and VCAM1 in patients with co-occurrence of OLP and HT in comparison with healthy controls by ELISA. (C) Relative expression of CXCL12, CXCL10, and VCAM1 mRNA were detected using qPCR in OLP patients in comparison with healthy controls. *P* < .05 as being statistically significant. ELISA = enzyme-linked immunosorbent assay, HT = Hashimoto thyroiditis, OLP = oral lichen planus, qPCR = quantitative real‑time PCR.

The top 3 genes (CXCL12, CXCL10, and VCAM1) were verified by ELISA assay. The results showed that the expression levels of CXCL12, CXCL10, and VCAM1 in the OLP and HT group were significantly higher than control group (Fig. 5A). It is worth noting that the expression levels of CXCL12, CXCL10, and VCAM1 were significantly higher than the control group in OLP and HT coexisting cases (Fig. 5B). To further validate our analysis, the mRNA of CXCL12, CXCL10, and VCAM1 of OLP samples in OLP and HT coexisting cases were determined. The results showed the mRNA expression levels of CXCL12, CXCL10, and VCAM1 in OLP and HT coexisting cases were significantly higher than control group (Fig. 5C). Therefore, it was concluded that the expression level of key genes increased may be an overlapping pathogenesis between OLP and HT, which might cause co-occurrence.

## 4. Discussion

OLP is a chronic inflammatory oral mucosal disorder mediated by T cells, with a multifactorial etiology, and HT is a common autoimmune disease characterized by hypothyroidism.^[22]^ Previous study reported a close relationship between OLP and HT,^[17]^ but possible mechanism behind this association warrants further investigation. Thus, estimating markers and associations between OLP and HT are thus of interest and may be novel therapeutic targets for OLP and HT.

In our analysis, we screened out 814 DEGs in OLP and 529 DEGs in HT, and analyzed these DEGs through a variety of bioinformatics analysis methods. The previous study detected expression profile of OLP and HT patients and their controls, but only focused on genes that were individually differentially expressed.^[23,24]^ In our study, We investigated co-expressed genes to understand relationships between OLP and HT and reveal potential biomarkers of HT-related OLP and the molecular biological mechanisms underlying them. In our results, we found that the most enriched pathways were cytokine signaling in immune system, which contained 17 genes, in all 76 co-DEGs. The histopathological characteristics of OLP and HT suggested that the cell-mediated immune response, including lymphocyte infiltration and activation of T lymphocytes, plays an important role in their pathogenesis.^[7,25]^ Therefore, the results of our analysis are further to prove the occurrence of both diseases involves immune-related pathological processes.

Next, to study the co-DEGs interaction relationship, we constructed a PPI network and screened out the modules with the highest scores in MCODE analysis, including CXCL12, CXCL10,VCAM1, CXCL9, MMP9, STAT1, SELL, CD163, IL7R, and CCL19. The top 10 genes expression increased in OLP and HT, which indicated these up-regulated genes are highly related to co-occurrence of both diseases. In Reactome database, these genes are involved inflammatory and immune response, mainly including NF-kappa-B signaling and MAPK6/MAPK4 signaling. Chemokines are proinflammatory cytokines, which play a pivotal role in the selective recruitment of T cells via chemokine receptors.^[26]^ Numerous previous studies have shown that chemokines, including CXCL10,^[27]^ CXCL12,^[28]^ CCL5 and CXCL9^[29]^ are implicated in the pathogenesis of OLP and HT. VCAM1 was originally identified as a cell adhesion molecule that helps regulate inflammation-associated vascular adhesion and the transendothelial migration of leukocytes, such as macrophages and T cells.^[30]^ In line with our results, recent evidence suggests that VCAM1 is closely associated with the progression of various immunological disorders, including rheumatoid arthritis, asthma, transplant rejection,^[31]^ OLP,^[32]^ and HT.^[32]^

Based on our analysis results, CIBERSORT algorithm was used to confirm the relationship between the VCAM1 expression and immune infiltration. Several immune cells, including macrophages M0, mast cells resting were noted to be significantly different between the OLP and control. Among infiltrating lymphocytes, T cells gamma delta and macrophages M0 were positively correlated with VCAM1 expression, whereas Plasma cells were negatively associated with VCAM1 expression in OLP samples. Regulatory T cell, T cell CD4 naïve, monocytes, T cells follicular helper, T cells CD4 memory resting and macrophages M1 were noted to be significantly different between the HT and control. Among these cells, macrophages M1, T cells follicular helper, and T cells CD4 naive were positively correlated with VCAM1 expression, whereas monocytes, T cells CD4 memory resting and Mast cells resting were negatively associated with VCAM1 expression. These results suggested that VCAM1 might modulate co-occurrence of OLP and HT by acting on these immune cells. However, the exact relationship between VCAM1 and these immune cells and the exact effect of VCAM1 on OLP and HT immune infiltration must be confirmed with further studies.

To further validate our analysis results, we verified the top 3 genes (CXCL12, CXCL10, and VCAM1) through ELISA and qPCR. Consistent with the results of the bioinformatics analysis, a significant elevation of CXCL12, CXCL10, and VCAM1 in OLP and HT patients than in control. The expression levels of CXCL12 and CXCL10 did not change significantly, when compared the coexistence of OLP and HT with the existence of OLP or HT alone. While, VCAM1 were upregulated significantly. The result indicated that the high expression of VCAM1 has a cumulative effect in patients with both OLP and HT, which VCAM1 may be a key target to reveal the co-occurrence mechanism of OLP and HT.

The present study puts forth some important insights into the common or overlapping pathogenesis of OLP and HT from the perspective of bioinformatics. First, microarray and bioinformatics analyses were applied to analyze whole transcriptome changes between control and OLP or HT, and identified the OLP-DEGs and HT-DEGs. Second, co-DEGs were identified by crossing the OLP-DEGs and HT-DEGs, and co-DEGs were functionally enriched by bioinformatics methods. Third, PPI network revealed crucial co-DEGs, which may reveal molecular biological mechanism of co-occurrence of OLP and HT. Fourth, the relationship between VCAM1 expression and immune infiltration was confirmed. Finally, we conducted ELISA and qPCR analysis to validate the results of our bioinformatics analysis, which reduced the false-positive rate of bioinformatics methods. In our study, we utilized data extracted from publicly available databases to identify key biomarkers and immune infiltration in the co-occurrence of OLP and HT. However, the dataset used did not specify whether the patients had hepatitis C virus infection. As a result, our analysis does not account for the potential confounding effect of hepatitis C virus infection. We acknowledge this limitation in the interpretation of our findings, as this factor was not included in the dataset.

## 5. Conclusion

We believe the data presented in this study strongly support the conclusions drawn regarding the co-occurrence of OLP and HT. Our bioinformatics analysis identified 76 co-DEGs that were significantly associated with immune pathways, particularly cytokine signaling and regulation of cell adhesion, which are crucial to the pathogenesis of both diseases. Furthermore, the PPI network analysis revealed that key genes such as VCAM1, CXCL12, and CXCL10 play a pivotal role in the immune response, which was consistent across both disease conditions. In addition, we performed further analyses using the CIBERSORT algorithm to evaluate immune cell infiltration in OLP and HT samples, confirming that immune infiltration, particularly related to VCAM1 expression, is a critical factor in the co-occurrence of these diseases. The findings were supported by experimental validation using ELISA and qPCR, which showed significantly elevated levels of CXCL12, CXCL10, and VCAM1 in patient samples compared to healthy controls. These results reinforce the hypothesis that immune-related pathways and key immune markers are integral to the pathogenesis of OLP and HT, thus strongly supporting the conclusions drawn in this study.

## Author contributions

**Conceptualization**: Hui Feng, Li Xiao.

**Data curation**: Hui Feng.

**Formal analysis**: Zhen Chen.

**Investigation**: Hui Feng, Zhen Chen.

**Methodology**: Hui Feng, Zhen Chen.

**Validation**: Hui Feng.

**Writing – original draft**: Hui Feng.

## Supplementary Material
