# Supplementary material for: Identification of key biomarkers and immune infiltration in the co-occurrence of oral lichen planus and Hashimoto’s thyroiditis by integrated bioinformatics analysis
Source: Medicine (Baltimore). 2026 May 12;104(49):e46324. doi: 10.1097/MD.0000000000046324 (PMC12688925; doi:10.1097/MD.0000000000046324)
Supplement: Supplementary file 1 [file medi-104-e46324-s001.docx]

**Supplementary Table 1 Enrichment Analyses of co-expression DEGs in the Metascape**

| Category | Description | LogP | Symbols |
| --- | --- | --- | --- |
| Reactome Gene Sets | Cytokine Signaling in  Immune system | -11.70058474 | BIRC3,APP,CCND1,BST2,HLA-DQB1,HLA-  DRB4,HMOX1,IL7R,CXCL10,IRS1,LTB,MAOA,MMP 9,CCL19,STAT1,VCAM1,ISG15,C1QB,GJA1,FABP4,H CLS1,LUM |
| Reactome Gene Sets | Cytokine Signaling in  Immune system | -11.70058474 | BIRC3,APP,CCND1,BST2,HLA-DQB1,HLA-  DRB4,HMOX1,IL7R,CXCL10,IRS1,LTB,MAOA,MMP 9,CCL19,STAT1,VCAM1,ISG15 |
| Reactome Gene Sets | Signaling by Interleukins | -7.650454218 | APP,CCND1,HMOX1,IL7R,CXCL10,IRS1,MAOA,MM P9,CCL19,STAT1,VCAM1 |
| Reactome Gene Sets | Interleukin-4 and Interleukin-13 signaling | -6.488853814 | CCND1,HMOX1,MAOA,MMP9,STAT1,VCAM1 |
| WikiPathways | Spinal cord injury | -6.218427089 | CCND1,C1QB,GJA1,CXCL10,LTB,MMP9 |
| WikiPathways | Photodynamic therapy- induced NF-kB survival signaling | -5.73808476 | BIRC3,CCND1,MMP9,VCAM1 |
| Canonical Pathways | PID FRA PATHWAY | -5.638900255 | CCND1,GJA1,HMOX1,MMP9 |
| Canonical Pathways | PIDAP1 PATHWAY | -4.523715515 | CCND1,FABP4,GJA1,MMP9 |
| KEGG Pathway | TNF signaling pathway | -3.727553298 | BIRC3,CXCL10,MMP9,VCAM1 |
| KEGG Pathway | Proteoglycans in cancer | -2.743145652 | CCND1,HCLS1,LUM,MMP9 |
| GO Biological  Processes | regulation of cell adhesion | -10.01547853 | CYP1B1,HLA-DQB1,HLA-  DRB4,HES1,TNC,IL7R,IDO1,EPCAM,SERPINE2,CCL 19,CXCL12,SFRP1,ST6GAL1,TGFBI,TPM1,VCAM1,B IRC3,FABP4,HCLS1,CXCL10,CXCL9,MT1X,STAT1,A POE,CCN2,HMOX1,BST2,MYH11 |
| GO Biological  Processes | regulation of cell adhesion | -10.01547853 | CYP1B1,HLA-DQB1,HLA-  DRB4,HES1,TNC,IL7R,IDO1,EPCAM,SERPINE2,CCL 19,CXCL12,SFRP1,ST6GAL1,TGFBI,TPM1,VCAM1 |
| GO Biological  Processes | cellular response to  cytokine stimulus | -8.604174475 | BIRC3,CYP1B1,FABP4,HCLS1,HES1,IL7R,CXCL10,C XCL9,MT1X,CCL19,CXCL12,SFRP1,STAT1,VCAM1 |
| GO Biological  Processes | cellular response to tumor necrosis factor | -7.777929633 | BIRC3,CYP1B1,FABP4,HES1,CCL19,SFRP1,STAT1,V CAM1 |
| GO Biological  Processes | response to tumor necrosis factor | -7.351044525 | BIRC3,CYP1B1,FABP4,HES1,CCL19,SFRP1,STAT1,V CAM1 |
| GO Biological  Processes | regulation of cell activation | -6.696797135 | APOE,CCN2,HLA-DQB1,HLA-  DRB4,HMOX1,HES1,IL7R,IDO1,SERPINE2,CCL19,SF RP1,VCAM1 |
| GO Biological  Processes | regulation of cell-cell  adhesion | -6.480123836 | HLA-DQB1,HLA-  DRB4,HES1,IL7R,IDO1,EPCAM,SERPINE2,CCL19,C XCL12,VCAM1 |
| GO Biological  Processes | positive regulation of cell adhesion | -5.543752405 | HLA-DQB1,HLA-  DRB4,HES1,IL7R,CCL19,CXCL12,SFRP1,TPM1,VCA |

|  |  |  | M1 |
| --- | --- | --- | --- |
| GO Biological  Processes | regulation of leukocyte cell- cell adhesion | -5.418851903 | HLA-DQB1,HLA-  DRB4,HES1,IL7R,IDO1,CCL19,CXCL12,VCAM1 |
| GO Biological  Processes | positive regulation of T cell activation | -4.457749109 | HLA-DQB1,HLA-DRB4,HES1,IL7R,CCL19,VCAM1 |
| GO Biological  Processes | regulation of T cell  activation | -4.427485544 | HLA-DQB1,HLA-  DRB4,HES1,IL7R,IDO1,CCL19,VCAM1 |
| GO Biological  Processes | regulation of leukocyte  activation | -4.410925214 | HLA-DQB1,HLA-  DRB4,HMOX1,HES1,IL7R,IDO1,CCL19,SFRP1,VCA M1 |
| GO Biological  Processes | regulation of leukocyte  proliferation | -4.341194462 | BST2,HES1,IDO1,CCL19,ST6GAL1,VCAM1 |
| GO Biological  Processes | positive regulation of  leukocyte proliferation | -4.28465316 | BST2,HES1,CCL19,ST6GAL1,VCAM1 |
| GO Biological  Processes | positive regulation of leukocyte cell-cell adhesion | -4.24858848 | HLA-DQB1,HLA-DRB4,HES1,IL7R,CCL19,VCAM1 |
| GO Biological  Processes | regulation of lymphocyte activation | -4.097261437 | HLA-DQB1,HLA-  DRB4,HES1,IL7R,IDO1,CCL19,SFRP1,VCAM1 |
| GO Biological  Processes | positive regulation of receptor signaling pathway via JAK-STAT | -3.916189936 | CYP1B1,HES1,IL7R |
| GO Biological  Processes | positive regulation of cell- cell adhesion | -3.859812026 | HLA-DQB1,HLA-DRB4,HES1,IL7R,CCL19,VCAM1 |
| GO Biological  Processes | positive regulation of cell activation | -3.805477998 | CCN2,HLA-DQB1,HLA-  DRB4,HES1,IL7R,CCL19,VCAM1 |
| GO Biological  Processes | positive regulation of receptor signaling pathway via STAT | -3.785526325 | CYP1B1,HES1,IL7R |
| GO Biological  Processes | regulation of mononuclear cell proliferation | -3.500181645 | HES1,IDO1,CCL19,ST6GAL1,VCAM1 |
| GO Biological  Processes | positive regulation of  lymphocyte activation | -3.335283861 | HLA-DQB1,HLA-DRB4,HES1,IL7R,CCL19,VCAM1 |
| GO Biological  Processes | positive regulation of  mononuclear cell  proliferation | -3.312064978 | HES1,CCL19,ST6GAL1,VCAM1 |
| GO Biological  Processes | positive regulation of  leukocyte activation | -3.062703844 | HLA-DQB1,HLA-DRB4,HES1,IL7R,CCL19,VCAM1 |
| GO Biological  Processes | regulation of receptor signaling pathway via JAK- STAT | -3.025167609 | CYP1B1,HES1,IL7R |
| GO Biological  Processes | regulation of T cell  proliferation | -2.995595844 | HES1,IDO1,CCL19,VCAM1 |
| GO Biological  Processes | regulation of receptor  signaling pathway via | -2.855441864 | CYP1B1,HES1,IL7R |

|  | STAT |  |  |
| --- | --- | --- | --- |
| GO Biological  Processes | positive regulation of T cell proliferation | -2.63368654 | HES1,CCL19,VCAM1 |
| GO Biological  Processes | regulation of lymphocyte proliferation | -2.555567011 | HES1,IDO1,CCL19,VCAM1 |
| GO Biological  Processes | cardiocyte differentiation | -2.553476166 | HES1,MYH11,VCAM1 |
| GO Biological  Processes | positive regulation of lymphocyte proliferation | -2.254120103 | HES1,CCL19,VCAM1 |
| GO Biological  Processes | response to inorganic  substance | -9.231566738 | APP,CCND1,CYP1B1,FABP4,HMOX1,MMP9,MT1G, MT1H,MT1X,SERPINF1,CCL19,STAT1,VCAM1,APO E,CXCL12,GLRX,RGS2,HOPX,PTGDS |
| GO Biological  Processes | response to inorganic  substance | -9.231566738 | APP,CCND1,CYP1B1,FABP4,HMOX1,MMP9,MT1G, MT1H,MT1X,SERPINF1,CCL19,STAT1,VCAM1 |
| GO Biological  Processes | response to metal ion | -7.717757762 | APP,CCND1,FABP4,HMOX1,MMP9,MT1G,MT1H,MT 1X,SERPINF1,VCAM1 |
| GO Biological  Processes | cellular response to metal ion | -7.557276705 | APP,FABP4,HMOX1,MMP9,MT1G,MT1H,MT1X,SER PINF1 |
| GO Biological  Processes | cellular response to  cadmium ion | -7.441849746 | HMOX1,MMP9,MT1G,MT1H,MT1X |
| GO Biological  Processes | cellular response to  inorganic substance | -7.065107084 | APP,FABP4,HMOX1,MMP9,MT1G,MT1H,MT1X,SER PINF1 |
| WikiPathways | Copper homeostasis | -6.595667359 | APP,CCND1,MT1G,MT1H,MT1X |
| GO Biological  Processes | response to cadmium ion | -6.400428176 | HMOX1,MMP9,MT1G,MT1H,MT1X |
| GO Biological  Processes | cellular response to copper ion | -6.2061823 | APP,MT1G,MT1H,MT1X |
| GO Biological  Processes | cellular metal ion  homeostasis | -5.873498072 | APOE,APP,HMOX1,MT1G,MT1H,MT1X,CCL19,CXC L12 |
| GO Biological  Processes | cellular divalent inorganic cation homeostasis | -5.849874825 | APOE,APP,MT1G,MT1H,MT1X,CCL19,CXCL12 |
| Reactome Gene Sets | Metallothioneins bind  metals | -5.603604253 | MT1G,MT1H,MT1X |
| GO Biological  Processes | divalent inorganic cation homeostasis | -5.459460081 | APOE,APP,MT1G,MT1H,MT1X,CCL19,CXCL12 |
| GO Biological  Processes | response to copper ion | -5.456570462 | APP,MT1G,MT1H,MT1X |
| Reactome Gene Sets | Response to metal ions | -5.262349344 | MT1G,MT1H,MT1X |
| GO Biological  Processes | cellular chemical  homeostasis | -5.189761895 | APOE,APP,HMOX1,MT1G,MT1H,MT1X,SERPINF1,C CL19,CXCL12 |
| GO Biological  Processes | cellular cation homeostasis | -5.167253054 | APOE,APP,HMOX1,MT1G,MT1H,MT1X,CCL19,CXC L12 |
| GO Biological | detoxification of copper ion | -5.166226691 | MT1G,MT1H,MT1X |

| Processes |  |  |  |
| --- | --- | --- | --- |
| GO Biological  Processes | stress response to copper ion | -5.166226691 | MT1G,MT1H,MT1X |
| GO Biological  Processes | metal ion homeostasis | -5.127404265 | APOE,APP,HMOX1,MT1G,MT1H,MT1X,CCL19,CXC L12 |
| GO Biological  Processes | cellular transition metal ion homeostasis | -5.094445966 | APP,HMOX1,MT1G,MT1H,MT1X |
| GO Biological  Processes | cellular ion homeostasis | -5.049359096 | APOE,APP,HMOX1,MT1G,MT1H,MT1X,CCL19,CXC L12 |
| GO Biological  Processes | response to zinc ion | -5.005372058 | MT1G,MT1H,MT1X,VCAM1 |
| GO Biological  Processes | detoxification of inorganic compound | -4.914909557 | MT1G,MT1H,MT1X |
| GO Biological  Processes | stress response to metal ion | -4.841062997 | MT1G,MT1H,MT1X |
| KEGG Pathway | Mineral absorption | -4.789674238 | HMOX1,MT1G,MT1H,MT1X |
| GO Biological  Processes | detoxification | -4.671570122 | APOE,GLRX,MT1G,MT1H,MT1X |
| GO Biological  Processes | transition metal ion  homeostasis | -4.609149904 | APP,HMOX1,MT1G,MT1H,MT1X |
| GO Biological  Processes | response to toxic substance | -4.601985168 | APOE,CYP1B1,GLRX,MT1G,MT1H,MT1X |
| GO Biological  Processes | cellular homeostasis | -4.585797054 | APOE,APP,HMOX1,MT1G,MT1H,MT1X,SERPINF1,C CL19,CXCL12 |
| GO Biological  Processes | cellular response to zinc ion | -4.525110428 | MT1G,MT1H,MT1X |
| GO Biological  Processes | cation homeostasis | -4.518715089 | APOE,APP,HMOX1,MT1G,MT1H,MT1X,CCL19,CXC L12 |
| GO Biological  Processes | inorganic ion homeostasis | -4.462213025 | APOE,APP,HMOX1,MT1G,MT1H,MT1X,CCL19,CXC L12 |
| GO Biological  Processes | ion homeostasis | -4.35864507 | APOE,APP,HMOX1,MT1G,MT1H,MT1X,CCL19,CXC L12 |
| GO Biological  Processes | cellular zinc ion  homeostasis | -4.024059774 | MT1G,MT1H,MT1X |
| WikiPathways | Zinc homeostasis | -3.95111987 | MT1G,MT1H,MT1X |
| GO Biological  Processes | zinc ion homeostasis | -3.916189936 | MT1G,MT1H,MT1X |
| GO Biological  Processes | cellular calcium ion  homeostasis | -2.906457116 | APOE,APP,CCL19,CXCL12 |
| GO Biological  Processes | regulation of  developmental growth | -2.835078699 | APOE,APP,RGS2,CXCL12,HOPX |
| GO Biological  Processes | calcium ion homeostasis | -2.720181781 | APOE,APP,CCL19,CXCL12 |

| GO Biological  Processes | myeloid leukocyte  activation | -2.288506924 | APP,MT1G,PTGDS |
| --- | --- | --- | --- |
| GO Biological  Processes | response to peptide | -8.965869219 | APP,CCN2,CYP1B1,GJA1,IRS1,MMP9,MYO5A,SERPI NF1,CXCL12,STAT1,VCAM1,LDOC1,CCND1,HCLS1, HMOX1,HES1,CCL19,SFRP1,IDO1,LTB,LUM,PDE4B, ISG15 |
| GO Biological  Processes | response to peptide | -8.965869219 | APP,CCN2,CYP1B1,GJA1,IRS1,MMP9,MYO5A,SERPI NF1,CXCL12,STAT1,VCAM1,LDOC1 |
| GO Biological  Processes | response to hormone | -7.329739865 | CCND1,CCN2,CYP1B1,HCLS1,HMOX1,HES1,IRS1,M YO5A,SERPINF1,CCL19,CXCL12,SFRP1,STAT1 |
| GO Biological  Processes | positive regulation of  cytokine production | -6.446987746 | APP,CYP1B1,HMOX1,IDO1,LTB,LUM,PDE4B,CCL19, STAT1,ISG15 |
| GO Biological  Processes | cellular response to peptide | -5.988423448 | APP,CYP1B1,GJA1,IRS1,MYO5A,STAT1,VCAM1,LD OC1 |
| GO Biological  Processes | cellular response to organonitrogen compound | -5.713220515 | APP,CYP1B1,GJA1,IRS1,MYO5A,PDE4B,SFRP1,STAT 1,VCAM1,LDOC1 |
| GO Biological  Processes | cellular response to  nitrogen compound | -5.334721171 | APP,CYP1B1,GJA1,IRS1,MYO5A,PDE4B,SFRP1,STAT 1,VCAM1,LDOC1 |
| GO Biological  Processes | response to peptide  hormone | -3.564310556 | CCN2,CYP1B1,IRS1,MYO5A,CXCL12,STAT1 |
| GO Biological  Processes | cellular response to  hormone stimulus | -2.869509606 | CYP1B1,IRS1,MYO5A,SERPINF1,SFRP1,STAT1 |
| GO Biological  Processes | response to cAMP | -2.827049959 | APP,CYP1B1,STAT1 |
| GO Biological  Processes | cellular response to organic cyclic compound | -2.762537768 | APP,CYP1B1,PDE4B,SERPINF1,SFRP1,STAT1 |
| GO Biological  Processes | cellular response to peptide hormone stimulus | -2.5091007 | CYP1B1,IRS1,MYO5A,STAT1 |
| GO Biological  Processes | response to  organophosphorus | -2.398605109 | APP,CYP1B1,STAT1 |
| GO Biological  Processes | response to purine- containing compound | -2.271178782 | APP,CYP1B1,STAT1 |
| WikiPathways | Endoderm differentiation | -2.220781049 | APP,SFRP1,STAT1 |
| GO Biological  Processes | cellular response to insulin stimulus | -2.196429823 | IRS1,MYO5A,STAT1 |
| GO Biological  Processes | negative regulation of leukocyte apoptotic process | -8.519492232 | HCLS1,IL7R,IDO1,CCL19,CXCL12,ST6GAL1,SFRP1, STAT1,ISG15,APOE,RGS2 |
| GO Biological  Processes | negative regulation of leukocyte apoptotic process | -8.519492232 | HCLS1,IL7R,IDO1,CCL19,CXCL12,ST6GAL1 |
| GO Biological  Processes | regulation of leukocyte apoptotic process | -7.110015685 | HCLS1,IL7R,IDO1,CCL19,CXCL12,ST6GAL1 |
| GO Biological  Processes | regulation of hemopoiesis | -3.283330934 | HCLS1,IL7R,CCL19,SFRP1,STAT1,ISG15 |
| GO Biological | regulation of leukocyte | -2.125584705 | HCLS1,IL7R,CCL19,SFRP1 |

| Processes | differentiation |  |  |
| --- | --- | --- | --- |
| GO Biological  Processes | regulation of anatomical structure size | -2.08302588 | APOE,HCLS1,IL7R,RGS2,CXCL12 |
| GO Biological  Processes | regulation of growth | -7.969959587 | APOE,APP,BST2,GJA1,TNC,MT1G,MT1H,MT1X,SER PINE2,RGS2,CXCL12,SFRP1,HOPX |
| GO Biological  Processes | regulation of growth | -7.969959587 | APOE,APP,BST2,GJA1,TNC,MT1G,MT1H,MT1X,SER PINE2,RGS2,CXCL12,SFRP1,HOPX |
| GO Biological  Processes | negative regulation of  growth | -6.707171918 | BST2,GJA1,MT1G,MT1H,MT1X,SERPINE2,RGS2,SF RP1 |
| GO Biological  Processes | regulation of cell growth | -4.97342775 | APOE,BST2,GJA1,TNC,SERPINE2,RGS2,CXCL12,SF RP1 |
| GO Biological  Processes | negative regulation of cell growth | -3.962923218 | BST2,GJA1,SERPINE2,RGS2,SFRP1 |
| WikiPathways | Malignant pleural  mesothelioma | -7.796934442 | CCND1,CDH11,CCN2,FABP4,IDO1,CXCL10,MMP9,S ERPINF1,CXCL12,SFRP1,STAT1 |
| WikiPathways | Malignant pleural  mesothelioma | -7.796934442 | CCND1,CDH11,CCN2,FABP4,IDO1,CXCL10,MMP9,S ERPINF1,CXCL12,SFRP1,STAT1 |
| WikiPathways | Hippo-Merlin signaling  dysregulation | -3.571747803 | CCND1,CDH11,CCN2,CXCL10 |
| GO Biological  Processes | extracellular matrix  organization | -7.554931443 | APP,CCN2,CTSK,CYP1B1,LUM,MMP9,MYH11,TGFB I,OLFML2A |
| GO Biological  Processes | extracellular matrix  organization | -7.554931443 | APP,CCN2,CTSK,CYP1B1,LUM,MMP9,MYH11,TGFB I,OLFML2A |
| GO Biological  Processes | extracellular structure  organization | -7.541188639 | APP,CCN2,CTSK,CYP1B1,LUM,MMP9,MYH11,TGFB I,OLFML2A |
| GO Biological  Processes | external encapsulating structure organization | -7.513865954 | APP,CCN2,CTSK,CYP1B1,LUM,MMP9,MYH11,TGFB I,OLFML2A |
| GO Biological  Processes | response to bacterium | -7.472784149 | ACP5,FABP4,JCHAIN,IL7R,IDO1,CXCL10,LYZ,CXCL 9,PDE4B,RGS1,VCAM1,ISG15,LDOC1,CYP1B1,HES1 ,IRS1,SERPINF1,SFRP1 |
| GO Biological  Processes | response to bacterium | -7.472784149 | ACP5,FABP4,JCHAIN,IL7R,IDO1,CXCL10,LYZ,CXCL 9,PDE4B,RGS1,VCAM1,ISG15,LDOC1 |
| GO Biological  Processes | cellular response to lipid | -5.312583992 | CYP1B1,HES1,CXCL10,IRS1,CXCL9,PDE4B,SERPIN F1,SFRP1,LDOC1 |
| GO Biological  Processes | response to  lipopolysaccharide | -4.801951518 | ACP5,IDO1,CXCL10,CXCL9,PDE4B,VCAM1,LDOC1 |
| GO Biological  Processes | response to molecule of bacterial origin | -4.623971471 | ACP5,IDO1,CXCL10,CXCL9,PDE4B,VCAM1,LDOC1 |
| GO Biological  Processes | negative regulation of cell population proliferation | -7.113895942 | APOE,APP,CYP1B1,HMOX1,HES1,IDO1,SERPINF1,S ERPINE2,SFRP1,STAT1,TPM1,WARS1,LDOC1,BST2, GJA1,CXCL12,ST6GAL1,CXCL10,RGS2,CCN2,TGFB I,APOC1,MAOA,PDE4B |
| GO Biological  Processes | negative regulation of cell population proliferation | -7.113895942 | APOE,APP,CYP1B1,HMOX1,HES1,IDO1,SERPINF1,S ERPINE2,SFRP1,STAT1,TPM1,WARS1,LDOC1 |

| GO  Processes | Biological | negative regulation of  locomotion | -7.046899774 | APOE,BST2,CYP1B1,GJA1,HMOX1,SERPINF1,CXCL 12,SFRP1,ST6GAL1,TPM1 |
| --- | --- | --- | --- | --- |
| GO  Processes | Biological | negative regulation of cell migration | -6.467698686 | APOE,BST2,CYP1B1,GJA1,HMOX1,SERPINF1,CXCL 12,SFRP1,TPM1 |
| GO  Processes | Biological | negative regulation of cell motility | -6.321196737 | APOE,BST2,CYP1B1,GJA1,HMOX1,SERPINF1,CXCL 12,SFRP1,TPM1 |
| GO  Processes | Biological | negative regulation of cellular component movement | -6.24565381 | APOE,BST2,CYP1B1,GJA1,HMOX1,SERPINF1,CXCL 12,SFRP1,TPM1 |
| GO  Processes | Biological | blood circulation | -5.072545924 | APOE,GJA1,HMOX1,CXCL10,RGS2,CXCL12,STAT1, TPM1 |
| GO  Processes | Biological | blood vessel  morphogenesis | -4.958486161 | APOE,CCN2,CYP1B1,GJA1,HMOX1,HES1,TGFBI,WA RS1 |
| GO  Processes | Biological | plasma lipoprotein particle clearance | -4.525110428 | APOC1,APOE,HMOX1 |
| GO  Processes | Biological | circulatory system process | -4.443641097 | APOE,GJA1,HMOX1,CXCL10,RGS2,CXCL12,STAT1, TPM1 |
| GO  Processes | Biological | bloodvessel development | -4.39473948 | APOE,CCN2,CYP1B1,GJA1,HMOX1,HES1,TGFBI,WA RS1 |
| GO  Processes | Biological | tube morphogenesis | -4.365027069 | APOE,CCN2,CYP1B1,GJA1,HMOX1,HES1,SFRP1,TG FBI,WARS1 |
| GO  Processes | Biological | vasculature development | -4.2705092 | APOE,CCN2,CYP1B1,GJA1,HMOX1,HES1,TGFBI,WA RS1 |
| GO  Processes | Biological | negative regulation of cell activation | -3.660330203 | APOE,HMOX1,IDO1,SERPINE2,SFRP1 |
| GO  Processes | Biological | regulation of plasma lipoprotein particle levels | -3.436789213 | APOC1,APOE,HMOX1 |
| GO  Processes | Biological | negative regulation of smooth muscle cell proliferation | -3.076012203 | APOE,HMOX1,TPM1 |
| GO  Processes | Biological | regulation of endothelial cell proliferation | -2.968277663 | APOE,HMOX1,CXCL12,STAT1 |
| GO  Processes | Biological | angiogenesis | -2.870235139 | CCN2,CYP1B1,HMOX1,TGFBI,WARS1 |
| GO  Processes | Biological | organic cyclic compound catabolic process | -2.603018594 | APOE,HMOX1,IDO1,MAOA,PDE4B |
| GO  Processes | Biological | response to nutrient | -7.094707047 | CCND1,CYP1B1,HMOX1,CXCL10,SFRP1,STAT1,VC AM1,GJA1,HES1,TNC,ID4,SERPINE2,LDOC1,APOE, CXCL12,APP,MMP9,SERPINF1,CCL19,CCN2,PDE4B, TLE2,CPE |
| GO  Processes | Biological | response to nutrient | -7.094707047 | CCND1,CYP1B1,HMOX1,CXCL10,SFRP1,STAT1,VC AM1 |
| GO  Processes | Biological | gland development | -6.108555424 | CCND1,CYP1B1,GJA1,HMOX1,HES1,TNC,ID4,SERPI NE2,SFRP1 |

| GO Biological  Processes | reproductive structure  development | -5.925864602 | CCND1,CYP1B1,GJA1,HES1,TNC,ID4,SERPINE2,SFR P1,LDOC1 |
| --- | --- | --- | --- |
| GO Biological  Processes | reproductive system  development | -5.900591582 | CCND1,CYP1B1,GJA1,HES1,TNC,ID4,SERPINE2,SFR P1,LDOC1 |
| GO Biological  Processes | response to nutrient levels | -4.77820764 | APOE,CCND1,CYP1B1,HMOX1,CXCL10,SFRP1,STA T1,VCAM1 |
| GO Biological  Processes | response to extracellular  stimulus | -4.557054797 | APOE,CCND1,CYP1B1,HMOX1,CXCL10,SFRP1,STA T1,VCAM1 |
| GO Biological  Processes | regeneration | -4.419096145 | CCND1,GJA1,HMOX1,TNC,CXCL12 |
| GO Biological  Processes | response to radiation | -3.943230035 | APP,CCND1,CXCL10,MMP9,CXCL12,SFRP1,VCAM1 |
| GO Biological  Processes | response to ketone | -3.877277727 | CCND1,CYP1B1,SERPINF1,CCL19,SFRP1 |
| GO Biological  Processes | response to estradiol | -3.571747803 | CCND1,CCN2,CYP1B1,SFRP1 |
| GO Biological  Processes | male gonad development | -3.405857061 | CCND1,CYP1B1,GJA1,SFRP1 |
| GO Biological  Processes | development of primary male sexual characteristics | -3.393811401 | CCND1,CYP1B1,GJA1,SFRP1 |
| GO Biological  Processes | response to ionizing  radiation | -3.346560995 | CCND1,CXCL10,SFRP1,VCAM1 |
| GO Biological  Processes | response to xenobiotic  stimulus | -3.277641884 | CCND1,CYP1B1,HMOX1,PDE4B,SFRP1,STAT1 |
| GO Biological  Processes | animal organ regeneration | -3.223226524 | CCND1,HMOX1,CXCL12 |
| GO Biological  Processes | response to estrogen | -3.147360973 | CCND1,HMOX1,SFRP1 |
| GO Biological  Processes | male sex differentiation | -3.140357415 | CCND1,CYP1B1,GJA1,SFRP1 |
| GO Biological  Processes | response to corticosteroid | -3.130253297 | CCND1,CCN2,CYP1B1,SERPINF1 |
| GO Biological  Processes | response to decreased  oxygen levels | -3.077963925 | CCN2,HMOX1,CXCL12,SFRP1,VCAM1 |
| KEGG Pathway | Wnt signaling pathway | -3.071032619 | CCND1,SERPINF1,SFRP1,TLE2 |
| GO Biological  Processes | response to vitamin | -3.008691206 | CCND1,CXCL10,SFRP1 |
| Canonical Pathways | PID BETACATENIN NUC PATHWAY | -2.960592443 | CCND1,MMP9,TLE2 |
| GO Biological  Processes | response to oxygen levels | -2.912210638 | CCN2,HMOX1,CXCL12,SFRP1,VCAM1 |
| Canonical Pathways | WNT SIGNALING | -2.827049959 | CCND1,SFRP1,TLE2 |
| WikiPathways | ncRNAs involved in Wnt | -2.799331064 | CCND1,SERPINF1,SFRP1 |

|  | signaling in hepatocellular carcinoma |  |  |
| --- | --- | --- | --- |
| GO Biological  Processes | cellular response to ketone | -2.707204221 | CYP1B1,SERPINF1,SFRP1 |
| GO Biological  Processes | positive regulation of epithelial cell proliferation | -2.705076666 | CCND1,HMOX1,CXCL12,SFRP1 |
| WikiPathways | lncRNA in canonical Wnt signaling and colorectal cancer | -2.63368654 | CCND1,SERPINF1,SFRP1 |
| GO Biological  Processes | gonad development | -2.631886776 | CCND1,CYP1B1,GJA1,SFRP1 |
| GO Biological  Processes | development of primary sexual characteristics | -2.596676448 | CCND1,CYP1B1,GJA1,SFRP1 |
| WikiPathways | Wnt signaling | -2.4785496 | CCND1,SERPINF1,SFRP1 |
| GO Biological  Processes | Wnt signaling pathway | -2.360812916 | CCND1,CPE,SFRP1,TLE2 |
| GO Biological  Processes | cell-cell signaling by wnt | -2.360812916 | CCND1,CPE,SFRP1,TLE2 |
| GO Biological  Processes | sex differentiation | -2.281304998 | CCND1,CYP1B1,GJA1,SFRP1 |
| GO Biological  Processes | response to steroid hormone | -2.281304998 | CCND1,CCN2,CYP1B1,SERPINF1 |
| GO Biological  Processes | response to glucocorticoid | -2.279808682 | CCND1,CYP1B1,SERPINF1 |
| GO Biological  Processes | response to hypoxia | -2.27580406 | HMOX1,CXCL12,SFRP1,VCAM1 |
| GO Biological  Processes | negative regulation of  canonical Wnt signaling  pathway | -2.254120103 | APOE,SFRP1,TLE2 |
| GO Biological  Processes | cell surface receptor signaling pathway involved in cell-cell signaling | -2.031354679 | CCND1,CPE,SFRP1,TLE2 |
| GO Biological  Processes | negative regulation of Wnt signaling pathway | -2.012465368 | APOE,SFRP1,TLE2 |
| WikiPathways | Network map of SARS- CoV-2 signaling pathway | -7.049965466 | APOC1,BST2,CXCL10,CXCL9,CXCL12,STAT1,CD163 ,IFI44L,ISG15,CCL19,CTSK,HCLS1,SFRP1,C1QB,GJA 1,ST6GAL1 |
| WikiPathways | Network map of SARS- CoV-2 signaling pathway | -7.049965466 | APOC1,BST2,CXCL10,CXCL9,CXCL12,STAT1,CD163 ,IFI44L |
| WikiPathways | Type II interferon signaling | -5.638900255 | CXCL10,CXCL9,STAT1,ISG15 |
| GO Biological  Processes | response to virus | -5.471939056 | BST2,CXCL10,CXCL9,CCL19,CXCL12,STAT1,ISG15, IFI44L |
| GO Biological  Processes | defense response to virus | -4.389010379 | BST2,CXCL10,CXCL9,STAT1,ISG15,IFI44L |

| GO Biological  Processes | defense response to  symbiont | | -4.379363491 | BST2,CXCL10,CXCL9,STAT1,ISG15,IFI44L |
| --- | --- | --- | --- | --- |
| KEGG Pathway | Toll-like receptor signaling pathway | | -3.851524428 | CTSK,CXCL10,CXCL9,STAT1 |
| WikiPathways | Non-genomic actions of 1,25 dihydroxyvitamin D3 | | -3.111154521 | STAT1,ISG15,IFI44L |
| Reactome Gene Sets | Interferon alpha/beta  signaling | | -3.093454108 | BST2,STAT1,ISG15 |
| GO Biological  Processes | negative regulation of viral process | | -2.799331064 | BST2,STAT1,ISG15 |
| GO Biological  Processes | regulation of myeloid cell differentiation | | -2.705076666 | HCLS1,SFRP1,STAT1,ISG15 |
| GO Biological  Processes | positive regulation of myeloid cell differentiation | | -2.682173649 | HCLS1,STAT1,ISG15 |
| WikiPathways | Toll-like receptor signaling pathway | | -2.63368654 | CXCL10,CXCL9,STAT1 |
| KEGG Pathway | Coronavirus disease -  COVID-19 | | -2.548832415 | C1QB,CXCL10,STAT1,ISG15 |
| Reactome Gene Sets | SARS-CoV-2 Infection | | -2.232604979 | GJA1,ST6GAL1,STAT1,ISG15 |
| GO Biological  Processes | regulation of viral process | | -2.119005083 | BST2,STAT1,ISG15 |
| WikiPathways | Allograft rejection | | -6.99004424 | C1QB,HLA-  DQB1,CXCL9,CCL19,CXCL12,STAT1,CXCL10,MMP9 ,PDE4B,SELL,VCAM1,CTSK,LTB,SERPINF1,SERPIN E2,SFRP1,APP,RGS1,BIRC3,IL7R,GLRX,HES1,RGS2, HMOX1,ISG15,CYP1B1,FABP4,IDO1,LDOC1,ST6GA L1,IRS1,ADGRL2 |
| WikiPathways | Allograft rejection | | -6.99004424 | C1QB,HLA-DQB1,CXCL9,CCL19,CXCL12,STAT1 |
| GO Biological  Processes | leukocyte migration | | -5.721586891 | CXCL10,CXCL9,MMP9,PDE4B,CCL19,SELL,VCAM1 |
| Canonical Pathways | NABA MATRISOME ASSOCIATED | | -5.55958152 | C1QB,CTSK,CXCL10,LTB,CXCL9,MMP9,SERPINF1, SERPINE2,CCL19,CXCL12,SFRP1 |
| GO Biological  Processes | cell chemotaxis | | -4.849304143 | CXCL10,CXCL9,PDE4B,CCL19,CXCL12,VCAM1 |
| Reactome Gene Sets | Chemokine receptors bind chemokines | | -4.818810611 | CXCL10,CXCL9,CCL19,CXCL12 |
| Reactome Gene Sets | G alpha (i) signalling events | | -4.801951518 | APP,CXCL10,CXCL9,PDE4B,RGS1,CCL19,CXCL12 |
| WikiPathways | Overview  proinflammatory  profibrotic mediators | of and | -4.719728212 | CXCL10,CXCL9,MMP9,CCL19,CXCL12 |
| WikiPathways | SARS-CoV-2 innate immunity evasion and cell- specific immune response | | -4.573552514 | CXCL10,CXCL9,CXCL12,STAT1 |
| GO Biological | cytokine-mediated | | -4.457888302 | BIRC3,IL7R,CXCL10,CXCL9,CCL19,CXCL12,STAT1 |

| Processes | signaling pathway |  |  |
| --- | --- | --- | --- |
| GO Biological  Processes | neutrophil chemotaxis | -4.31665587 | CXCL10,CXCL9,PDE4B,CCL19 |
| GO Biological  Processes | chemokine-mediated signaling pathway | -4.253143615 | CXCL10,CXCL9,CCL19,CXCL12 |
| GO Biological  Processes | granulocyte chemotaxis | -4.212154873 | CXCL10,CXCL9,PDE4B,CCL19 |
| WikiPathways | Chemokine signaling  pathway | -4.208294313 | CXCL10,CXCL9,CCL19,CXCL12,STAT1 |
| GO Biological  Processes | positive regulation of T cell migration | -4.184095887 | APP,CXCL10,CXCL12 |
| GO Biological  Processes | neutrophil migration | -4.114048831 | CXCL10,CXCL9,PDE4B,CCL19 |
| WikiPathways | Prostaglandin signaling | -4.101523295 | CXCL10,CXCL9,MMP9 |
| GO Biological  Processes | regulation of metal ion  transport | -4.09618267 | GLRX,HES1,CXCL10,CXCL9,PDE4B,SERPINE2,CXC L12 |
| GO Biological  Processes | response to chemokine | -4.095130253 | CXCL10,CXCL9,CCL19,CXCL12 |
| GO Biological  Processes | cellular response to  chemokine | -4.095130253 | CXCL10,CXCL9,CCL19,CXCL12 |
| KEGG Pathway | Cytokine-cytokine receptor interaction | -3.992407008 | IL7R,CXCL10,LTB,CXCL9,CCL19,CXCL12 |
| GO Biological  Processes | granulocyte migration | -3.968647328 | CXCL10,CXCL9,PDE4B,CCL19 |
| GO Biological  Processes | positive regulation of  lymphocyte migration | -3.95111987 | APP,CXCL10,CXCL12 |
| KEGG Pathway | Viral protein interaction with cytokine and cytokine receptor | -3.917383937 | CXCL10,CXCL9,CCL19,CXCL12 |
| KEGG Pathway | Chemokine signaling  pathway | -3.89832309 | CXCL10,CXCL9,CCL19,CXCL12,STAT1 |
| Reactome Gene Sets | Peptide ligand-binding  receptors | -3.805442598 | APP,CXCL10,CXCL9,CCL19,CXCL12 |
| Reactome Gene Sets | GPCR downstream  signalling | -3.716432043 | APP,CXCL10,CXCL9,PDE4B,RGS1,RGS2,CCL19,CXC L12 |
| GO Biological  Processes | regulation of T cell  migration | -3.695841294 | APP,CXCL10,CXCL12 |
| Canonical Pathways | NABA SECRETED  FACTORS | -3.637711174 | CXCL10,LTB,CXCL9,CCL19,CXCL12,SFRP1 |
| GO Biological  Processes | regulation of leukocyte  migration | -3.632638018 | APP,HMOX1,CXCL10,CCL19,CXCL12 |
| GO Biological  Processes | myeloid leukocyte  migration | -3.545030448 | CXCL10,CXCL9,PDE4B,CCL19 |

| GO Biological  Processes | chemotaxis | | -3.542185834 | APP,CXCL10,CXCL9,PDE4B,CCL19,CXCL12,VCAM1 |
| --- | --- | --- | --- | --- |
| GO Biological  Processes | taxis | | -3.531855169 | APP,CXCL10,CXCL9,PDE4B,CCL19,CXCL12,VCAM1 |
| Reactome Gene Sets | Signaling by GPCR | | -3.390662039 | APP,CXCL10,CXCL9,PDE4B,RGS1,RGS2,CCL19,CXC L12 |
| GO Biological  Processes | positive regulation of  leukocyte migration | | -3.346560995 | APP,CXCL10,CCL19,CXCL12 |
| GO Biological  Processes | regulation of calcium ion transport | | -3.306705712 | HES1,CXCL10,CXCL9,PDE4B,CXCL12 |
| WikiPathways | Novel intracellular components of RIG-I-like receptor (RLR) pathway | | -3.304192688 | CXCL10,CXCL12,ISG15 |
| GO Biological  Processes | leukocyte chemotaxis | | -3.30073897 | CXCL10,CXCL9,PDE4B,CCL19 |
| GO Biological  Processes | positive regulation of cell migration | | -3.278956156 | APP,CYP1B1,HMOX1,CXCL10,MMP9,CCL19,CXCL1 2 |
| GO Biological  Processes | regulation of lymphocyte migration | | -3.242964653 | APP,CXCL10,CXCL12 |
| GO Biological  Processes | positive regulation of mononuclear cell migration | | -3.203806599 | APP,CXCL10,CXCL12 |
| GO Biological  Processes | positive regulation of cell motility | | -3.171492229 | APP,CYP1B1,HMOX1,CXCL10,MMP9,CCL19,CXCL1 2 |
| GO Biological  Processes | positive regulation of cellular component movement | | -3.115474892 | APP,CYP1B1,HMOX1,CXCL10,MMP9,CCL19,CXCL1 2 |
| GO Biological  Processes | positive regulation of  locomotion | | -3.10698755 | APP,CYP1B1,HMOX1,CXCL10,MMP9,CCL19,CXCL1 2 |
| GO Biological  Processes | positive response stimulus | regulation of to external | -2.992811343 | APP,FABP4,IDO1,CXCL10,CCL19,CXCL12 |
| Reactome Gene Sets | Class A/1 (Rhodopsin-like receptors) | | -2.800652338 | APP,CXCL10,CXCL9,CCL19,CXCL12 |
| GO Biological  Processes | cellular response to  lipopolysaccharide | | -2.782262496 | CXCL10,CXCL9,PDE4B,LDOC1 |
| GO Biological  Processes | positive regulation of  leukocyte chemotaxis | | -2.758950371 | CXCL10,CCL19,CXCL12 |
| GO Biological  Processes | response to mechanical  stimulus | | -2.758663737 | CXCL10,SERPINE2,CXCL12,STAT1 |
| GO Biological  Processes | response to heat | | -2.707204221 | HMOX1,CXCL10,CXCL12 |
| GO Biological  Processes | cellular response to molecule of bacterial origin | | -2.690131212 | CXCL10,CXCL9,PDE4B,LDOC1 |
| GO Biological | regulation of chemotaxis | | -2.589739834 | CXCL10,CCL19,CXCL12,ST6GAL1 |

| Processes |  |  |  |
| --- | --- | --- | --- |
| GO Biological  Processes | positive regulation of transmembrane transport | -2.575969559 | GLRX,CXCL10,IRS1,CXCL9 |
| GO Biological  Processes | adenylate cyclase- modulating G protein- coupled receptor signaling pathway | -2.569135291 | CXCL10,CXCL9,RGS1,ADGRL2 |
| GO Biological  Processes | cellular response to biotic stimulus | -2.502588842 | CXCL10,CXCL9,PDE4B,LDOC1 |
| GO Biological  Processes | regulation of mononuclear cell migration | -2.488954418 | APP,CXCL10,CXCL12 |
| GO Biological  Processes | regulation of leukocyte  chemotaxis | -2.418063236 | CXCL10,CCL19,CXCL12 |
| GO Biological  Processes | regulation of sequestering of calcium ion | -2.408291838 | CXCL10,CXCL9,CCL19 |
| GO Biological  Processes | positive regulation of  calcium ion transport | -2.408291838 | CXCL10,CXCL9,CXCL12 |
| GO Biological  Processes | positive regulation of  chemotaxis | -2.271178782 | CXCL10,CCL19,CXCL12 |
| GO Biological  Processes | positive regulation of ion transport | -2.270325958 | GLRX,CXCL10,CXCL9,CXCL12 |
| GO Biological  Processes | adenylate cyclase- activating G protein- coupled receptor signaling pathway | -2.262616242 | CXCL10,CXCL9,ADGRL2 |
| Reactome Gene Sets | GPCR ligand binding | -2.186542296 | APP,CXCL10,CXCL9,CCL19,CXCL12 |
| GO Biological  Processes | positive regulation of cation transmembrane transport | -2.082291292 | GLRX,CXCL10,CXCL9 |
| GO Biological  Processes | response to temperature  stimulus | -2.046802043 | HMOX1,CXCL10,CXCL12 |
| KEGG Pathway | Rheumatoid arthritis | -6.87565727 | ACP5,CTSK,HLA-DQB1,HLA-  DRB4,LTB,CXCL12,BST2,STAT1,VCAM1,ISG15,CPE, BIRC3,TUBB2A,CCND1,THBS2,CXCL10,C1QB,IL7R, CD79A,SELL,APP,PTGDS |
| KEGG Pathway | Rheumatoid arthritis | -6.87565727 | ACP5,CTSK,HLA-DQB1,HLA-DRB4,LTB,CXCL12 |
| Reactome Gene Sets | Interferon Signaling | -4.946404607 | BST2,HLA-DQB1,HLA-DRB4,STAT1,VCAM1,ISG15 |
| Reactome Gene Sets | Interferon gamma signaling | -4.076433547 | HLA-DQB1,HLA-DRB4,STAT1,VCAM1 |
| KEGG Pathway | Type I diabetes mellitus | -3.754903096 | CPE,HLA-DQB1,HLA-DRB4 |
| KEGG Pathway | Toxoplasmosis | -3.727553298 | BIRC3,HLA-DQB1,HLA-DRB4,STAT1 |
| KEGG Pathway | Intestinal immune network for IgA production | -3.585579325 | HLA-DQB1,HLA-DRB4,CXCL12 |
| Reactome Gene Sets | MHC class II antigen  presentation | -3.571747803 | CTSK,HLA-DQB1,HLA-DRB4,TUBB2A |

| KEGG Pathway | Viral myocarditis | -3.325310681 | CCND1,HLA-DQB1,HLA-DRB4 |
| --- | --- | --- | --- |
| KEGG Pathway | Phagosome | -3.223754603 | HLA-DQB1,HLA-DRB4,THBS2,TUBB2A |
| KEGG Pathway | Inflammatory bowel  disease | -3.223226524 | HLA-DQB1,HLA-DRB4,STAT1 |
| KEGG Pathway | Influenza A | -3.032830706 | HLA-DQB1,HLA-DRB4,CXCL10,STAT1 |
| KEGG Pathway | Leishmaniasis | -3.008691206 | HLA-DQB1,HLA-DRB4,STAT1 |
| KEGG Pathway | Th1 and Th2 cell  differentiation | -2.785714665 | HLA-DQB1,HLA-DRB4,STAT1 |
| KEGG Pathway | Staphylococcus aureus  infection | -2.732788599 | C1QB,HLA-DQB1,HLA-DRB4 |
| KEGG Pathway | Hematopoietic cell lineage | -2.694621127 | HLA-DQB1,HLA-DRB4,IL7R |
| KEGG Pathway | Th17 cell differentiation | -2.587164958 | HLA-DQB1,HLA-DRB4,STAT1 |
| Reactome Gene Sets | Adaptive Immune System | -2.491108807 | CD79A,CTSK,HLA-DQB1,HLA- DRB4,SELL,TUBB2A,VCAM1 |
| GO Biological  Processes | immune effector process | -2.318710665 | APP,C1QB,HLA-DQB1,HLA-DRB4,PTGDS |
| KEGG Pathway | Systemic lupus  erythematosus | -2.306112467 | C1QB,HLA-DQB1,HLA-DRB4 |
| GO Biological  Processes | leukocyte mediated  immunity | -2.281304998 | C1QB,HLA-DQB1,HLA-DRB4,PTGDS |
| KEGG Pathway | Herpes simplex virus 1  infection | -2.08302588 | BIRC3,BST2,HLA-DQB1,HLA-DRB4,STAT1 |
| GO Biological  Processes | immunoglobulin mediated immune response | -2.019243739 | C1QB,HLA-DQB1,HLA-DRB4 |
| GO Biological  Processes | negative regulation of  transport | -6.505179996 | APOC1,APOE,GJA1,HMOX1,HES1,IRS1,MMP9,SERP INE2,RGS2,SFRP1,CCND1,SERPINF1,CXCL12,STAT1 ,RCAN1,APP,FABP4,CCL19,WARS1,CXCL10,ISG15 |
| GO Biological  Processes | negative regulation of  transport | -6.505179996 | APOC1,APOE,GJA1,HMOX1,HES1,IRS1,MMP9,SERP INE2,RGS2,SFRP1 |
| GO Biological  Processes | regulation of epithelial cell proliferation | -5.265243026 | APOE,CCND1,HMOX1,HES1,SERPINF1,CXCL12,SF RP1,STAT1 |
| GO Biological  Processes | negative regulation of  secretion by cell | -4.461329758 | APOE,GJA1,HMOX1,IRS1,SFRP1 |
| GO Biological  Processes | negative regulation of  secretion | -4.146919772 | APOE,GJA1,HMOX1,IRS1,SFRP1 |
| GO Biological  Processes | negative regulation of intracellular signal transduction | -4.140875989 | APOE,RCAN1,MMP9,SERPINE2,RGS2,CXCL12,SFRP 1,STAT1 |
| GO Biological  Processes | regulation of kinase activity | -3.836662126 | APOE,APP,CCND1,FABP4,IRS1,RGS2,CCL19,SFRP1, WARS1 |
| GO Biological  Processes | negative regulation of  protein kinase activity | -3.68844897 | APOE,FABP4,RGS2,SFRP1,WARS1 |
| GO Biological | regulation of protein kinase | -3.594034177 | APOE,APP,CCND1,FABP4,RGS2,CCL19,SFRP1,WAR |

| Processes | activity |  | S1 |
| --- | --- | --- | --- |
| GO Biological  Processes | negative regulation of  kinase activity | -3.47481857 | APOE,FABP4,RGS2,SFRP1,WARS1 |
| GO Biological  Processes | negative regulation of MAP kinase activity | -3.325310681 | APOE,RGS2,SFRP1 |
| GO Biological  Processes | negative regulation of  hormone secretion | -3.304192688 | GJA1,IRS1,SFRP1 |
| GO Biological  Processes | regulation of  morphogenesis of an  epithelium | -3.223226524 | GJA1,CXCL10,SFRP1 |
| GO Biological  Processes | negative regulation of  transferase activity | -3.210124829 | APOE,FABP4,RGS2,SFRP1,WARS1 |
| GO Biological  Processes | positive regulation of lipid metabolic process | -3.181473905 | APOC1,APOE,IRS1,CCL19 |
| GO Biological  Processes | negative regulation of  protein secretion | -3.165882902 | APOE,IRS1,SFRP1 |
| GO Biological  Processes | regulation of neuron projection development | -3.103822902 | APOE,HES1,SERPINF1,RGS2,CXCL12,SFRP1 |
| GO Biological  Processes | negative regulation of epithelial cell proliferation | -3.100347046 | APOE,SERPINF1,SFRP1,STAT1 |
| GO Biological  Processes | negative regulation of phosphate metabolic process | -3.067795702 | APOC1,APOE,FABP4,RGS2,SFRP1,WARS1 |
| GO Biological  Processes | negative regulation of phosphorus metabolic process | -3.062703844 | APOC1,APOE,FABP4,RGS2,SFRP1,WARS1 |
| GO Biological  Processes | regulation of plasma membrane bounded cell projection organization | -2.999682545 | APOE,HES1,SERPINF1,RGS2,CCL19,CXCL12,SFRP1 |
| GO Biological  Processes | regulation of cell projection organization | -2.932419352 | APOE,HES1,SERPINF1,RGS2,CCL19,CXCL12,SFRP1 |
| GO Biological  Processes | regulation of MAP kinase activity | -2.923853694 | APOE,RGS2,CCL19,SFRP1 |
| GO Biological  Processes | negative regulation of protein phosphorylation | -2.772502514 | APOE,FABP4,RGS2,SFRP1,WARS1 |
| GO Biological  Processes | positive regulation of cell projection organization | -2.761376213 | APOE,SERPINF1,RGS2,CCL19,CXCL12 |
| GO Biological  Processes | negative regulation of  protein modification  process | -2.698731699 | APOE,FABP4,RGS2,SFRP1,WARS1,ISG15 |
| GO Biological  Processes | regulation of secretion by cell | -2.562006628 | APOE,GJA1,HMOX1,IRS1,CXCL12,SFRP1 |
| GO Biological  Processes | negative regulation of  phosphorylation | -2.55352154 | APOE,FABP4,RGS2,SFRP1,WARS1 |

| GO Biological  Processes | regulation of serine/threonine activity | protein kinase | -2.524548799 | APOE,CCND1,RGS2,CCL19,SFRP1 |
| --- | --- | --- | --- | --- |
| GO Biological  Processes | regulation of ossification | | -2.447899445 | GJA1,SFRP1,ISG15 |
| GO Biological  Processes | negative regulation of protein serine/threonine kinase activity | | -2.437865603 | APOE,RGS2,SFRP1 |
| GO Biological  Processes | negative regulation of  protein transport | | -2.418063236 | APOE,IRS1,SFRP1 |
| GO Biological  Processes | negative regulation of establishment of protein localization | | -2.379480283 | APOE,IRS1,SFRP1 |
| GO Biological  Processes | regulation of secretion | | -2.357853656 | APOE,GJA1,HMOX1,IRS1,CXCL12,SFRP1 |
| GO Biological  Processes | positive regulation of neuron projection development | | -2.172613353 | APOE,SERPINF1,RGS2 |
| GO Biological  Processes | ossification | | -6.350399551 | CDH11,CCN2,CTSK,GJA1,TNC,ID4,MMP9,SFRP1,AC P5,TGFBI,FAT1,HES1,TPM1,CCND1 |
| GO Biological  Processes | ossification | | -6.350399551 | CDH11,CCN2,CTSK,GJA1,TNC,ID4,MMP9,SFRP1 |
| GO Biological  Processes | prostate gland epithelium morphogenesis | | -4.582315693 | TNC,ID4,SFRP1 |
| GO Biological  Processes | prostate gland  morphogenesis | | -4.470379705 | TNC,ID4,SFRP1 |
| GO Biological  Processes | prostate gland development | | -3.754903096 | TNC,ID4,SFRP1 |
| GO Biological  Processes | skeletal system  development | | -3.547369154 | ACP5,CDH11,CCN2,GJA1,MMP9,SFRP1,TGFBI |
| GO Biological  Processes | osteoblast differentiation | | -3.455014342 | GJA1,TNC,ID4,SFRP1 |
| GO Biological  Processes | tissue morphogenesis | | -3.292775944 | FAT1,GJA1,HES1,TNC,ID4,SFRP1,TPM1 |
| GO Biological  Processes | morphogenesis of an  epithelium | | -3.032434752 | FAT1,GJA1,HES1,TNC,ID4,SFRP1 |
| WikiPathways | Vitamin D receptor  pathway | | -2.897836432 | CCND1,ID4,SFRP1,TPM1 |
| GO Biological  Processes | gland morphogenesis | | -2.657674711 | TNC,ID4,SFRP1 |
| GO Biological  Processes | negative regulation of  catalytic activity | | -6.221849726 | BIRC3,APOC1,APOE,APP,BST2,FABP4,MMP9,SERPI NF1,SERPINE2,RGS2,SFRP1,WARS1,CCN2,HMOX1, CTSK |
| GO Biological | negative regulation of | | -6.221849726 | BIRC3,APOC1,APOE,APP,BST2,FABP4,MMP9,SERPI |

| Processes | catalytic activity |  | NF1,SERPINE2,RGS2,SFRP1,WARS1 |
| --- | --- | --- | --- |
| GO Biological  Processes | negative regulation of  hydrolase activity | -5.358131413 | BIRC3,APOC1,APP,BST2,MMP9,SERPINF1,SERPINE 2,RGS2 |
| GO Biological  Processes | negative regulation of  endopeptidase activity | -4.379363491 | BIRC3,APP,BST2,MMP9,SERPINF1,SERPINE2 |
| GO Biological  Processes | negative regulation of  peptidase activity | -4.275959791 | BIRC3,APP,BST2,MMP9,SERPINF1,SERPINE2 |
| GO Biological  Processes | regulation of endopeptidase activity | -3.999429472 | BIRC3,APP,BST2,CCN2,MMP9,SERPINF1,SERPINE2 |
| GO Biological  Processes | regulation of peptidase  activity | -3.817136469 | BIRC3,APP,BST2,CCN2,MMP9,SERPINF1,SERPINE2 |
| GO Biological  Processes | negative regulation of  proteolysis | -3.577461655 | BIRC3,APP,BST2,MMP9,SERPINF1,SERPINE2 |
| Reactome Gene Sets | Nucleotide-binding  domain, leucine rich repeat containing receptor (NLR)  signaling pathways | -3.390964354 | BIRC3,APP,HMOX1 |
| GO Biological  Processes | regulation of proteolysis | -3.245034984 | BIRC3,APOE,APP,BST2,CCN2,MMP9,SERPINF1,SER PINE2 |
| Canonical Pathways | NABA ECM  REGULATORS | -2.5091007 | CTSK,MMP9,SERPINF1,SERPINE2 |
| Reactome Gene Sets | Toll-like Receptor  Cascades | -2.134050896 | BIRC3,APP,CTSK |
| GO Biological  Processes | hematopoietic or lymphoid organ development | -6.134438539 | APP,CD79A,HCLS1,HES1,IL7R,LTB,MMP9,MT1G,CC L19,SFRP1,VCAM1,BST2,GJA1,CXCL10,PTGDS |
| GO Biological  Processes | hematopoietic or lymphoid organ development | -6.134438539 | APP,CD79A,HCLS1,HES1,IL7R,LTB,MMP9,MT1G,CC L19,SFRP1,VCAM1 |
| GO Biological  Processes | immune system  development | -5.859661464 | APP,CD79A,HCLS1,HES1,IL7R,LTB,MMP9,MT1G,CC L19,SFRP1,VCAM1 |
| GO Biological  Processes | cell activation | -5.293237887 | APP,BST2,CD79A,GJA1,IL7R,CXCL10,MT1G,PTGDS, CCL19,VCAM1 |
| GO Biological  Processes | leukocyte activation | -5.15650034 | APP,BST2,CD79A,GJA1,IL7R,MT1G,PTGDS,CCL19,V CAM1 |
| GO Biological  Processes | hemopoiesis | -4.668913978 | APP,CD79A,HCLS1,IL7R,MMP9,MT1G,CCL19,SFRP1 ,VCAM1 |
| GO Biological  Processes | leukocyte differentiation | -4.473232244 | APP,CD79A,IL7R,MMP9,MT1G,CCL19,VCAM1 |
| GO Biological  Processes | lymphocyte activation | -3.188791924 | BST2,CD79A,GJA1,IL7R,CCL19,VCAM1 |
| GO Biological  Processes | mononuclear cell  differentiation | -3.044807614 | CD79A,IL7R,MT1G,CCL19,VCAM1 |
| GO Biological  Processes | B cell activation | -3.042288731 | BST2,CD79A,IL7R,VCAM1 |
| GO Biological | lymphocyte proliferation | -2.899361576 | CD79A,GJA1,IL7R |

| Processes |  |  |  |
| --- | --- | --- | --- |
| GO Biological  Processes | mononuclear cell  proliferation | -2.884538477 | CD79A,GJA1,IL7R |
| GO Biological  Processes | leukocyte proliferation | -2.719925745 | CD79A,GJA1,IL7R |
| GO Biological  Processes | regulation of inflammatory response | -6.126504909 | ACP5,BIRC3,APOE,APP,FABP4,TNC,IDO1,MMP9,SE RPINF1,STAT1,ISG15,HMOX1,HES1,LDOC1,HOPX,R CAN1,CXCL12,LYZ,TGFBI,IL7R,SERPINE2,CCND1, C1QB |
| GO Biological  Processes | regulation of inflammatory response | -6.126504909 | ACP5,BIRC3,APOE,APP,FABP4,TNC,IDO1,MMP9,SE RPINF1 |
| GO Biological  Processes | regulation of defense  response | -6.089330968 | ACP5,BIRC3,APOE,APP,FABP4,TNC,IDO1,MMP9,SE RPINF1,STAT1,ISG15 |
| GO Biological  Processes | regulation of binding | -4.40492883 | APOE,APP,HMOX1,HES1,MMP9,LDOC1,HOPX |
| GO Biological  Processes | locomotory behavior | -3.973910363 | APOE,APP,RCAN1,IDO1,CXCL12 |
| GO Biological  Processes | regulation of protein  binding | -3.846149835 | APOE,APP,MMP9,LDOC1,HOPX |
| Reactome Gene Sets | Amyloid fiber formation | -3.742523111 | APOE,APP,LYZ,TGFBI |
| WikiPathways | Apoptosis-related network due to altered Notch3 in ovarian cancer | -3.460364247 | APOE,APP,IL7R |
| GO Biological  Processes | behavior | -3.224548684 | APOE,APP,RCAN1,IDO1,SERPINF1,SERPINE2,CXCL 12 |
| GO Biological  Processes | positive regulation of  binding | -3.023433107 | APOE,APP,HES1,MMP9 |
| GO Biological  Processes | positive regulation of  protein binding | -2.899361576 | APOE,APP,MMP9 |
| Reactome Gene Sets | Post-translational protein  phosphorylation | -2.587164958 | APOE,APP,TNC |
| GO Biological  Processes | memory | -2.447899445 | APOE,RCAN1,SERPINF1 |
| Reactome Gene Sets | Regulation of Insulin-like Growth Factor (IGF) transport and uptake by Insulin-like Growth Factor Binding Proteins (IGFBPs) | -2.408291838 | APOE,APP,TNC |
| GO Biological  Processes | learning or memory | -2.343371607 | APOE,APP,RCAN1,SERPINF1 |
| WikiPathways | TGF-beta signaling  pathway | -2.333059149 | APP,CCND1,TNC |
| GO Biological  Processes | synapse organization | -2.211527955 | APOE,APP,C1QB,TNC |

| GO Biological  Processes | positive regulation of  inflammatory response | | -2.172613353 | APP,FABP4,IDO1 |
| --- | --- | --- | --- | --- |
| GO Biological  Processes | cognition | | -2.120703527 | APOE,APP,RCAN1,SERPINF1 |
| KEGG Pathway | Epstein-Barr virus infection | | -6.097509732 | CCND1,HLA-DQB1,HLA-  DRB4,HES1,CXCL10,STAT1,ISG15,CD79A,CCN2,GJ A1,HMOX1,ID4,IL7R,TGFBI,MMP9,BIRC3,CXCL12, TNC,THBS2,APP,IRS1,RCAN1,MYH11,VCAM1,TLE2, HOPX |
| KEGG Pathway | Epstein-Barr virus infection | | -6.097509732 | CCND1,HLA-DQB1,HLA-  DRB4,HES1,CXCL10,STAT1,ISG15 |
| GO Biological  Processes | cell population proliferation | | -5.39012444 | CCND1,CD79A,CCN2,GJA1,HMOX1,ID4,IL7R,STAT1 ,TGFBI |
| GO Biological  Processes | negative regulation of epithelial cell differentiation | | -5.107204641 | CCND1,HES1,MMP9,STAT1 |
| WikiPathways | IL-7 signaling pathway | | -4.470379705 | CCND1,IL7R,STAT1 |
| KEGG Pathway | Pathways in cancer | | -4.241775196 | BIRC3,CCND1,HMOX1,HES1,IL7R,MMP9,CXCL12,S TAT1 |
| KEGG Pathway | Human papillomavirus  infection | | -3.720891866 | CCND1,HES1,TNC,STAT1,THBS2,ISG15 |
| WikiPathways | DYRK1A | | -3.283436781 | APP,CCND1,HES1 |
| GO Biological  Processes | response to fatty acid | | -3.263031176 | CCN2,HES1,IRS1 |
| GO Biological  Processes | regulation of epithelial cell differentiation | | -3.171087984 | CCND1,HES1,MMP9,STAT1 |
| WikiPathways | VEGFA-VEGFR2 signaling pathway | | -3.072901155 | CCND1,CCN2,RCAN1,GJA1,MYH11,STAT1 |
| WikiPathways | Leptin signaling pathway | | -3.025167609 | CCND1,IRS1,STAT1 |
| WikiPathways | Acute viral myocarditis | | -2.855441864 | CCND1,MMP9,STAT1 |
| Reactome Gene Sets | Transcriptional regulation  by RUNX3 | | -2.732788599 | CCND1,CCN2,HES1 |
| KEGG Pathway | AGE-RAGE  pathway in complications | signaling diabetic | -2.682173649 | CCND1,STAT1,VCAM1 |
| Reactome Gene Sets | Signaling by NOTCH | | -2.522217708 | CCND1,HES1,STAT1,TLE2 |
| Reactome Gene Sets | Transcriptional regulation  by RUNX2 | | -2.447899445 | CCND1,HES1,STAT1 |
| KEGG Pathway | Thyroid hormone signaling pathway | | -2.447899445 | CCND1,RCAN1,STAT1 |
| GO Biological  Processes | liver development | | -2.437865603 | CCND1,HMOX1,HES1 |
| GO Biological  Processes | hepaticobiliary system  development | | -2.408291838 | CCND1,HMOX1,HES1 |

| GO Biological  Processes | positive regulation of  mitotic cell cycle | -2.37003958 | APP,CCND1,HES1 |
| --- | --- | --- | --- |
| KEGG Pathway | JAK-STAT signaling  pathway | -2.096825609 | CCND1,IL7R,STAT1 |
| GO Biological  Processes | lung development | -2.012465368 | CCN2,HES1,HOPX |
